# Predicting anti-SARS-CoV-2 activities of chemical compounds using machine learning models

**DOI:** 10.1016/j.aichem.2023.100029

**Published:** 2023-11-19

**Authors:** Beihong Ji, Yuhui Wu, Elena N. Thomas, Jocelyn N. Edwards, Xibing He, Junmei Wang

**Affiliations:** Department of Pharmaceutical Sciences and Computational Chemical Genomics Screening Center, School of Pharmacy, University of Pittsburgh, Pittsburgh, PA 15261, USA

**Keywords:** COVID-19, Machine Learning, In Silico Screening, Antiviral Compounds, SHAP Analysis, Attentive FP, Graph-Based Neural Networks

## Abstract

To accelerate the discovery of novel drug candidates for Coronavirus Disease 2019 (COVID-19) therapeutics, we reported a series of machine learning (ML)-based models to accurately predict the anti-SARS-CoV-2 activities of screening compounds. We explored 6 popular ML algorithms in combination with 15 molecular descriptors for molecular structures from 9 screening assays in the COVID-19 OpenData Portal hosted by NCATS. As a result, the models constructed by k-nearest neighbors (KNN) using the molecular descriptor GAFF+RDKit achieved the best overall performance with the highest average accuracy of 0.68 and relatively high average area under the receiver operating characteristic curve of 0.74, better than other ML algorithms. Meanwhile, The KNN model for all assays using GAFF+RDKit descriptor outperformed using other descriptors. The overall performance of our developed models was better than REDIAL-2020 (**R**). A web server (https://clickff.org/amberweb/covid-19-cp) was developed to enable users to predict anti-SARS-CoV-2 activities of arbitrary compounds using the COVID-19-CP (**P**) models. Besides the descriptor-based machine learning models, we also developed graph-based Attentive FP (**A**) models for the 9 assays. We found that the Attentive FP models achieved a comparable performance to that of COVID-19-CP and outperformed the REDIAL-2020 models. The consensus prediction utilizing both COVID-19-CP and Attentive FP can significantly boost the prediction accuracy as assessed by comparing its performance with other three individual models (**R**, **P**, **A**) utilizing the Wilcoxon signed-rank test, thus can ultimately improve the success rate of COVID-19 drug discovery.

## 1. Introduction

Since 2019, the Coronavirus Disease 2019 (COVID-19) pandemic has hit and put global health systems at risk [1]. So far, this novel coronavirus disease, caused by severe acute respiratory syndrome coronavirus 2 (SARS-CoV-2), has led to more than 757 million people infected with mortality reaching over 6.8 million as of 19 February 2023 [2]. Although the mortality and spread of COVID-19 has been suppressed due to rapidly increasing vaccination rates, there is still an urgent need for effective drug treatment for the large-scaling infection of this coronavirus. To speed up the identification of novel candidates for COVID-19 treatment in the drug discovery process, machine learning (ML) has stood out as a powerful tool for its efficiency and reliability in drug screenings [3–5].

In 2020, KC et al. [6] proposed a suite of ML-based models to predict activities of small molecules for SARS-CoV-2 from molecular structures related to several SARS-CoV-2 assays. The models they developed, coined “REDIAL-2020”, offered a convenient and efficient way to screen novel molecules for anti-SARS-CoV-2 activities. In this work, we further improved the performance of the prediction models by exploring different ML-based models and molecular descriptors regarding to molecular structures. Moreover, we created prediction models for three more screening assays. In totaFl six popular ML algorithms which include support vector machine (SVM) [7], logistic regression (LR) [8], decision tree (DT) [9], Random Forest (RF) [10], k-nearest neighbors (KNN) [11], and complement Naïve Bayes (CNB) [12,13], were applied to construct prediction models. A variety of molecular descriptors, including fingerprint (FP2, FP3, FP4 and MACCS), atom type counts (General AMBER Force Field (GAFF)), molecular properties (RDKit), were first applied to construct models. Four fingerprints translate various structural features into binary bits which are organized in a bit string with fixed length. RDKit collects 208 molecular properties while GAFF applies 47 atom types to describe subtle chemical environment. By combining different types of molecular descriptors, the hybrid ones which contain versatile structural information at different levels can enable us to construct high-quality models. We utilized 5 classification metrics as detailed in the supplemental text, to select combination of descriptors. In total, 9 pairs of sets of descriptors were exploited, including RDKit+FP2, RDKit+FP3, RDKit+FP4, RDKit+MACCS, GAFF+FP2, GAFF+FP3, GAFF+FP4, GAFF+MACCS, and GAFF+RDKit. We only consider the combination of molecular descriptors that are made of two-type descriptors to avoid redundant structural information and model complexity which decrease the model performance. All the 15 molecular descriptors were applied to train experimental screening data collected in the COVID-19 OpenData Portal (https://ncats.nih.gov/expertise/covid19-open-data-portal) hosted by National Center for Advancing Translational Sciences (NCATS). The 9 screening assays in this study can be classified into four categories, which are (1) viral replication, (2) live virus infectability, (3) viral entry, and (4) counter-screen. Both KC et al. and we studied the first six screening assays: 3CL enzymatic activity (3CL) in Category 1, SARS-CoV-2 cytopathic effect CPE in Category 2, Spike-ACE2 protein-protein interaction AlphaLISA assay and ACE2 enzymatic activity in Category 3, SARS-CoV-2 cytopathic effect counter-screen and Spike-ACE2 protein--protein interaction TruHit counter-screen in Category 4. Besides the above six assays, we also studied other three screening assays: TMPRSS2 enzymatic activity in Category 3, HEK 293 cell line toxicity and human fibroblast toxicity in Category 4.

For the only assay in Category 1, the papain-like proteinase 3CL cleaves SARS-CoV-2 polyprotein into individual proteins, which is a key process in the viral life cycle [14]. Inhibiting polyprotein cleavage can interrupt viral replication, making 3CL an attractive target in drug discovery and development. For assays in Category 2, the SARS-CoV-2 cytopathic effect (CPE) assay serves to measure the potential of compounds to reverse the cytopathic effect of the virus in Vero E6 host cells [15]. Thus, this assay can identify compounds with the potential to protect host cells from the CPE of the virus. Three assays belong to Category 3, measuring the ability of a compound inhibiting viral entry. The surface angiotensin-converting enzyme type 2 (ACE2) has been known as the primary host factor identified and targeted by SARS-CoV-2 virions [16,17]. The attachment of viral capsid to the host cell is facilitated by the SARS-CoV-2 Spike protein binding to the host ACE2, which trigger a multistep process of viral entry resulting in delivery of the viral genome to the cytosol, the site of replication. As a result, the disruption of the Spike-ACE2 interaction can cripple SARS-CoV-2 virions to infect host cells. The Spike-ACE2 protein-protein interaction AlphaLISA assay is used to measure the ability of therapeutics (small molecules, etc.) to disrupt the interaction between the Spike protein and ACE2. On the other hand, ACE2 plays a role in cleaving angiotensin I hormone into the vasoconstricting angiotensin II and acts as a counter-balance to ACE. Although inhibition of the Spike-ACE2 interaction can stop viral entry, off-target effects on endogenous ACE2 function may lead to disruption of critical vasodilation pathways. After the ACE2 binding, transmembrane protease serine 2 (TMPRSS2), a host protease which is essential for Spike protein priming, has been shown playing important a role in virus-host cell membrane fusion and further infection [18]. Therefore, the ACE2 and TMPRSS2 enzymatic assays can be applied to screen compounds with the ability to interrupt endogenous enzyme functions.

There are four assays in the counter-screen category. The counter-screen of the CPE assay is host cell tox counter-screen (Cytotox) and is used to measure cell cytotoxicity. Another counter-screen is the Spike-ACE2 protein-protein interaction TruHit assay that serves to identify false positives. The function of this assay is to investigate whether the activity found in a AlphaLISA assay is caused by the interference with the assay system itself or not. Two extra HEK293 cell line toxicity and human fibroblast toxicity assays are used as cell viability assays which evaluate the general human cell toxicity of compounds.

**Fig. 1.**
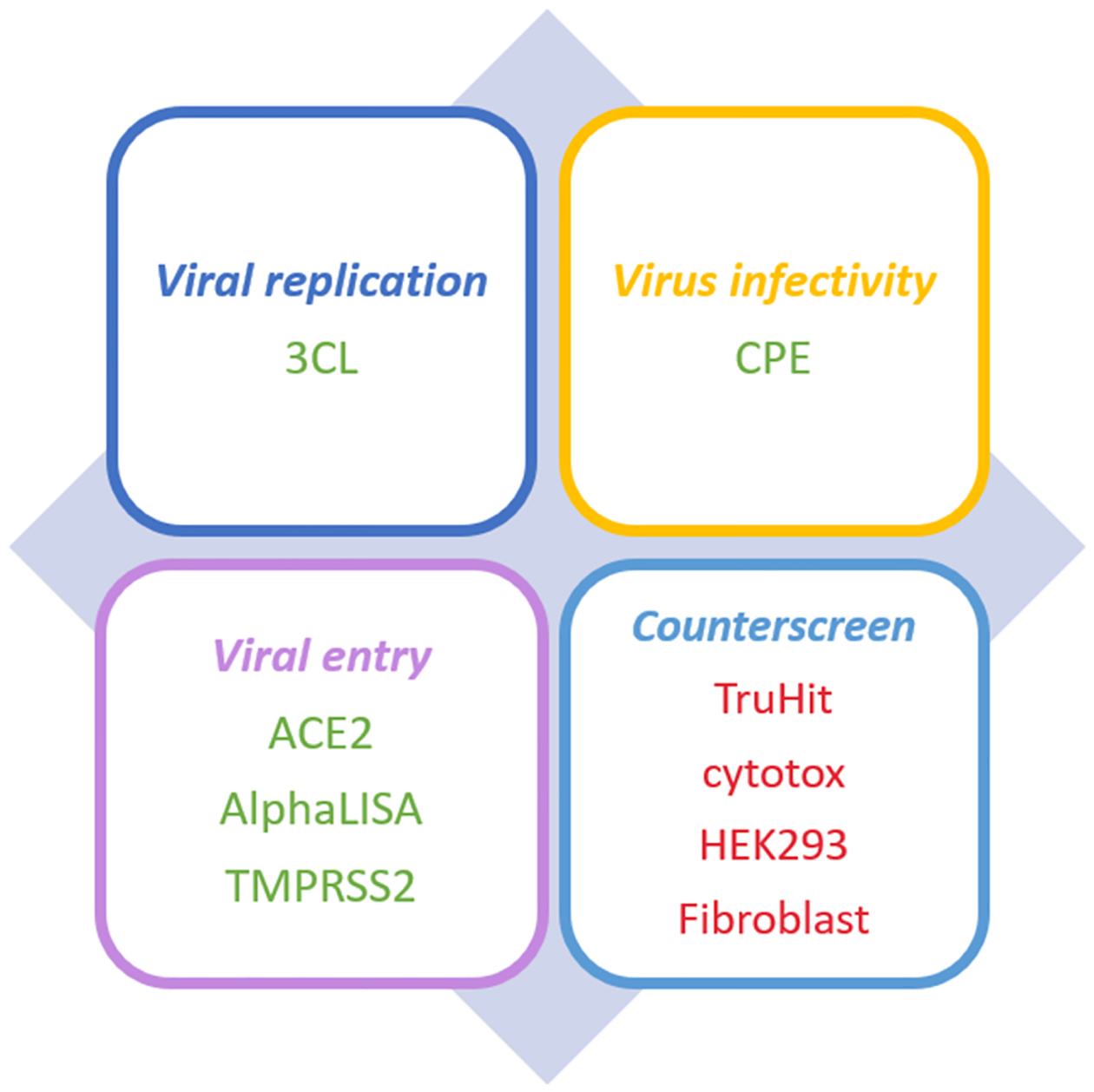
The desirable profile for the predicted anti-SARS-CoV-2 activities of the promising compound among 9 assays. Green color indicates “active” is preferred, while red color indicates “inactive” is preferred.

In Fig. 1, we illustrate the preferred response for each assay. For the assays in the first three categories, active response is preferred, while for the assays in the counter-screen category, we expect negative response which means no interference in the companion assays.

Next, we constructed a series of models by ML methods that can screen and identify compounds with anti-SARS-CoV-2 activities. The impacts of ML algorithms and molecular descriptors on the model performance regarding to different coronavirus-related assays were explored in this study. The model with the best performance was interpreted by the Shapley additive explanations (SHAP) values, from which the importance of descriptors and molecular features can be evaluated. In addition, the final satisfactory models were deployed in a web server with multiple molecular input formats, allowing a user to predict the activities of arbitrary small molecules against viral replication, viral entry and live virus infectivity. We believe the web server may provide users a convenient way to prioritize virtual screening drugs prior to in vitro or in vivo assays in rational drug discovery for pre-venting and treating COVID-19.

Nevertheless, other than using the traditional descriptor-based models, we adopted a novel graph-based modeling algorithm, Attentive FP [19], for the prediction of anti-SARS-CoV-2 activities of a compound. This graph neural network was proposed by Xiong, Z. et al. for molecular representation. It can not only characterize atomic local properties of a given chemical structure but also describe nonlocal intramolecular interactions. Attentive FP has been verified to achieve state-of-the-art predictive performances in the recent studies [19,20]. In this work, we are interested in finding out whether our descriptor-based models can achieve comparable or better accuracy to the more complicated graph-based neural networks models.

## 2. Methods

### 2.1. Data preparation and molecular representations

**Fig. 2.**
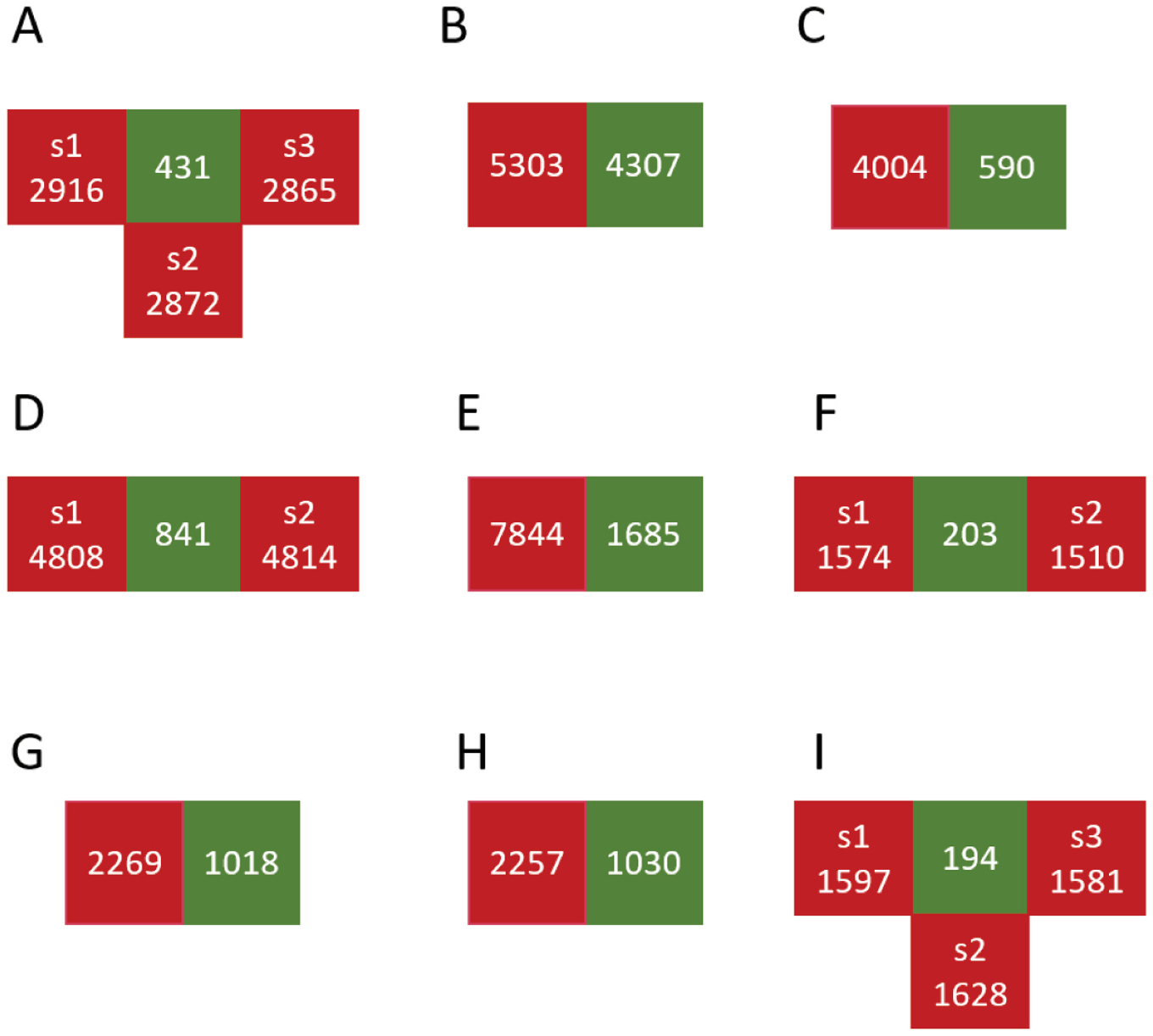
The counts of compounds in active sets (green) and inactive sets (red) for each assay. *s* refers to sample of inactive compounds (s1: sample 1, s2: sample 2, s3: sample 3, s4: sample 4). A-I are different assays. A. 3CL, B. HEK293, C. Fibroblast, D. CPE, E. Cytotox, F. ACE2, G. AlphaLISA, H. TruHit, I. TMPRSS2.

The compounds used for model training and testing for all assays were collected from the NCATS COVID-19 OpenData portal (https://opendata.ncats.nih.gov/covid19/). After removing duplicated compounds in each assay, we separated the compounds into active and inactive sets, based on whether the assay had half-maximal activity concentration (AC50) data. For some assays, the numbers of active and inactive are significantly unbalanced (for example, N_inactive_/N_active_ > 10), so we randomly divided inactive compounds into several subsets, and all subsets have similar numbers of inactives. We only used one subset of the inactive to construct models, and used the others as external test sets to evaluate the model performance. Specifically, 3CL has four subsets of inactives (s1, s2, s3, and s4), ACE2 has two, CPE has two, TMPRSS2 has three subsets of inactives. The distribution of active and inactive compounds in all assays is shown in Fig. 2. For the above assays, only the first subset (s1), was applied in the model construction.

To construct a machine learning model of an assay, we constructed a test dataset by randomly selecting 20% compounds in active set, and the same number of molecules from the inactive set. Note that numbers of actives and inactives in the training sets are unbalanced, therefore, we applied the RandomOverSampler (for 3CL assay) and SMOTE algorithms (for all other assays) [21] to overcome the data imbalance issue. Counts of active and inactive molecules in training (mean counts), validation (mean counts) sets and test sets for nine assays were summarized in Table 1.

As shown in the table, for most assays, the total compounds in the active and inactive sets are imbalanced. For the 3CL, CPE and Cytotox assays, the total inactive compounds are approximately five times greater than the actives. To resolve the issue of data imbalance, there are typically two resampling methods: under-sampling and oversampling [22]. Under-sampling refers to randomly removing some subjects from the majority class to match the counts of samples in the minority class. In the oversampling process, a sample of synthetic data for minority class was generated to match the number of samples in majority. Considering the dramatic difference of numbers of active and inactive compounds in some assays, we performed oversampling to resample the imbalanced data. We first applied class weight to balance the data, i.e., the under-sampled class had larger weight and the total weight of each class was roughly same. However, in that case, the calculated F1 scores of the models for most assays were unsatisfactory. For example, for the CPE assay, the average F1 score of all SVM models for test sets was only 0.42. Thus, we adopted another commonly used technique, Synthetic Minority Over-sampling Technique (SMOTE) implemented in a python program. SMOTE works by looking at examples that are close in the feature space for the minority class and draws a new sample at points along the line between the examples in the feature space [21]. By applying SMOTE technique, the average F1 score for the CPE assay under the same condition was improved to 0.55. We employed SMOTE package for almost all assays except for 3CL assay. Considering the complexity of the data in 3CL assay, we adopted a simpler RandomOverSampler [23] method to balance the data set.

Generally, the collected molecules were converted into three types of descriptors: i) fingerprint-based, ii) Physicochemical, iii) atom type-based. Class i) includes FP2 (1024 bits), FP3 (55 bits), FP4 (307 bits) and MACCS (166 bits). Among them, FP2 is a path-based fingerprint that indexes small molecule fragments based on linear segments of up to 7 atoms, while FP3, FP4 and MACCS are substructure-based fingerprints based on sets of SMILES arbitrary target specification (SMARTS) patterns. All fingerprint-based descriptors were obtained using Open Babel program version 2.3.1 (http://openbabel.org) [24]. RDKit molecular descriptor has 208 bits of vectors, belongs to class ii), and was obtained using RDKit program [25]. GAFF is an atom type-based molecular descriptor. It has 47 bits of vectors and GAFF contains molecular mechanics force field parameters for a wide breadth of molecules comprised of H, C, N, O, S, P and the halogens. In addition to the above six single descriptors, we also combined them to generate nine extra molecular descriptors: RDKit+FP2, RDKit+FP3, RDKit+FP4, RDKit+MACCS. GAFF+FP2, GAFF+FP3, GAFF+FP4, GAFF+MACCS, and GAFF+RDKit. Specifically, before the feature matrix served as the input data for ML-based models, its molecular descriptors containing RDKit features were standardized into matrix with values ranging from zero to one. This conversion was implemented utilizing *MinMaxScaler* in scikit-learn module.

### 2.2. Descriptor-based models

Several ML classifiers were constructed for each assay using 15 molecular descriptors and 6 ML algorithms. ML algorithms applied in the study include support vector machine (SVM), logistic regression (LR), decision tree (DT), Random Forest (RF), k-nearest neighbors (KNN) and complement Naïve Bayes (NB). The description and hyper-parameters of those ML algorithms are shown in supplemental information. For each assay, all models were trained and validated using partitioned data sets through the built classifiers in scikit-learn package of Python. The data in the separated test sets for each assay is then used for further model evaluation after the training.

### 2.3. Attentive FP model

Attentive FP (**A**) is a state-of-art graph-based neural network proposed by Xiong et al. [19] for the prediction of molecular characteristics. In their work, they introduced a graph attention mechanism which allows a method to focus on the relevant parts of the graph input to reach good prediction results. We fully implemented the code downloaded from GitHub (https://github.com/OpenDrugAI/AttentiveFP) and constructed **A** models. To objectively compare the performance of developed descriptor-based and **A** models, the same datasets used in the training and evaluation of descriptor-based models were adopted in the construction of graph-based models. The development of the **A** models was performed using the PyTorch package.

### 2.4. Model evaluation and performance metrics

The descriptor-based models were evaluated using stratified 10-fold cross-validation of the compounds excluded the test dataset and additionally tested by four external datasets. The cross validation was conducted by leveraging StratifiedKFold, a scikit-learn module built in python [26]. For a graph-based **A** model, we first conducted the same procedures we did for the descriptor-based models. For each assay, 20% compounds in active set and the same number of molecules from the inactive set were randomly extracted to comprise the test set. Next, to make sure that we completely integrated the built model, instead of using the python package to overcome the data imbalance and conducting cross-validation method for evaluation, we divided inactives into several sample sets in some assays to balance the ratio between the amounts of actives and inactives. For 3CL, CPE, Cytotox assays, the inactives were divided into 5 sample sets to match the amounts of actives. For ACE2, Fibroblast, TMPRSS2 assays, the inactives were divided into 10 sets. As such, the inactives in AlphaLISA and Truhit were divided into 2 sets. For HEK293 assay, the inactive compounds were not divided because the ratio of counts of actives and inactives was close to 1:1. Then for each assay, every divided inactive set and active set were combined to form a balanced dataset, thus, there were 5 balanced datasets compiled for 3CL, CPE, Cytotox assays, 10 balanced datasets for ACE2, Fibroblast, and TMPRSS2 assays, 2 balanced datasets for AlphaLISA and Truhit assays and 1 balanced dataset for HEK293 assay. Within each balanced set, the training and validation sets were randomly divided at a ratio of 9:1. The following-up model performance evaluation was performed by averaging the prediction results of all balanced sets in each assay.

The performance of all constructed models was evaluated by using five metrics: area under the curve (AUC) of receiver operating characteristic (ROC) curve, accuracy (ACC), F1-score, precision (PRE), and recall (REC). All of the metrics are ranged in [0,1], in which 0 indicates the worst and 1 indicates the best scenarios. Theoretically for AUC of ROC, a random model will have an AUC of 0.5. ACC measures the proportion of all correct cases among total evaluated cases. PRE is the measurement of the correct positive predictions from all predicted positive cases, while REC measures the correct positive predictions from all actual positive cases. F1-score is the harmonic mean of PRE and REC. The above five metrics were utilized to evaluate the performance of validation and test sets. Additionally, for the evaluation of external datasets, we employed sensitivity and specificity metrics to measure the model performance. Sensitivity, with the same meaning as recall, measures the percentage of compounds which received a positive prediction on this test out of the percentage of those which have the condition, whereas specificity measures the fraction of compounds which had a negative result on the test out of those which have no condition. Formulas of all metrics in the study are described in the supplemental information.

## 3. Results and discussion

### 3.1. Data set preparation

For the ACE2, Fibroblast and TMPRSS2 screening assays, the severity of data imbalance is very high, since for each assay the number of inactive compounds is even 10-fold larger than that of actives. Thus, we randomly divided inactive compounds for each of the four assays into 2–4 subsets based on the actual number of inactives. One inactive subset was randomly selected to participate in ML-based model construction, while the rest of sample sets were taken as external datasets for further model validation. Of note, the dataset consisted of all actives and inactives in the selected subset of inactives was still unbalanced.

### 3.2. ML-based model performance

For each screening assay collected in the COVID-19 open-data-portal, we constructed binary classification models with 15 different molecular descriptors using six different ML algorithms. The model performance measured by the validation and test sets under all conditions is presented in Table 2 and Figs. S1 and S2. Overall, the model performance measured by the validation sets was comparable for all assays (Table 2). Specifically, the KNN model stood out as it has higher scores of AUC (0.91) and REC (0.94), as well as comparable scores of ACC (0.80), F1(0.82) and PRE (0.73) compared to other ML methods. Meanwhile, the model performance measured by the test sets was slightly lower than that of the validation sets. The overall ranking for test sets of different machine learning models was the same, with the KNN method outperforming other ML models. The performance of KNN was generally satisfactory, which achieved the highest scores of ACC (0.68), F1 (0.69) and REC (0.71), and relatively high scores of AUC (0.74) and PRE (0.67) among all ML algorithms.

We then evaluated the performance of the KNN models constructed for all screening assays. Table 3 listed the average scores of metrics for all KNN models constructed using different molecular descriptors for individual assays. Essentially, there was no dramatic difference of those measured metrics among all the screening assays, indicating KNN was a promising ML algorithm to be applied to construct prediction models for screening data. Notably, for validation sets, even average scores of some metrics of the KNN models for most common assays were higher than the best scores reported in KC et al.’s study. For test sets, the average scores of some metrics, such as AUC for CPE, Cytotox and TruHit assays, were still higher than the best scores in KC et al.’s study. The scores of metrics for model evaluation that are better than those by KC et al. were highlighted with blue and bold font in Table 3. For example, the average AUC, ACC, F1, PRE and REC scores for test set for CPE assay is 0.75, 0.69, 0.71, 0.68, 0.74 respectively, which are much higher than the values in KC et al.’s model (0.651, 0.643, 0.661, 0.651, and 0.626 correspondingly).

In addition to the above evaluation metrics, sum of ranking differences (SRD) [27], a commonly used technique to resolve multicriteria optimization issues in many fields, was also considered as an additional measurement to compare the various ML algorithms. According to the previous publications [28],SRD is calculated using the actual data and serves as performance metric, similar to AUC or accuracy. In this work, SRD was utilized to compare the binary prediction results of six ML-based models across various assays. It was performed using the Python package on Github (https://github.com/davidbajusz/srdpy) [29]. According to Table 4, although KNN ranks in the middle of all six algorithms for the validation set, it ranks the best with the smallest SRD value for the test set. And if we compared the average SRD performance of six algorithms, KNN ranks second, and is only 0.16% smaller than RF. However, it has been reported that the performance metrics have different degrees of disagreement with SRD values for the classification problem, particularly for 2-class classification and imbalanced data sets [30]. Thus, SRD was not considered as the main metrics in this study, and instead, AUC and accuracy are more important performance metrics to evaluate the model performance.

### 3.3. Impact of molecular descriptor on model performance

**Fig. 3.**
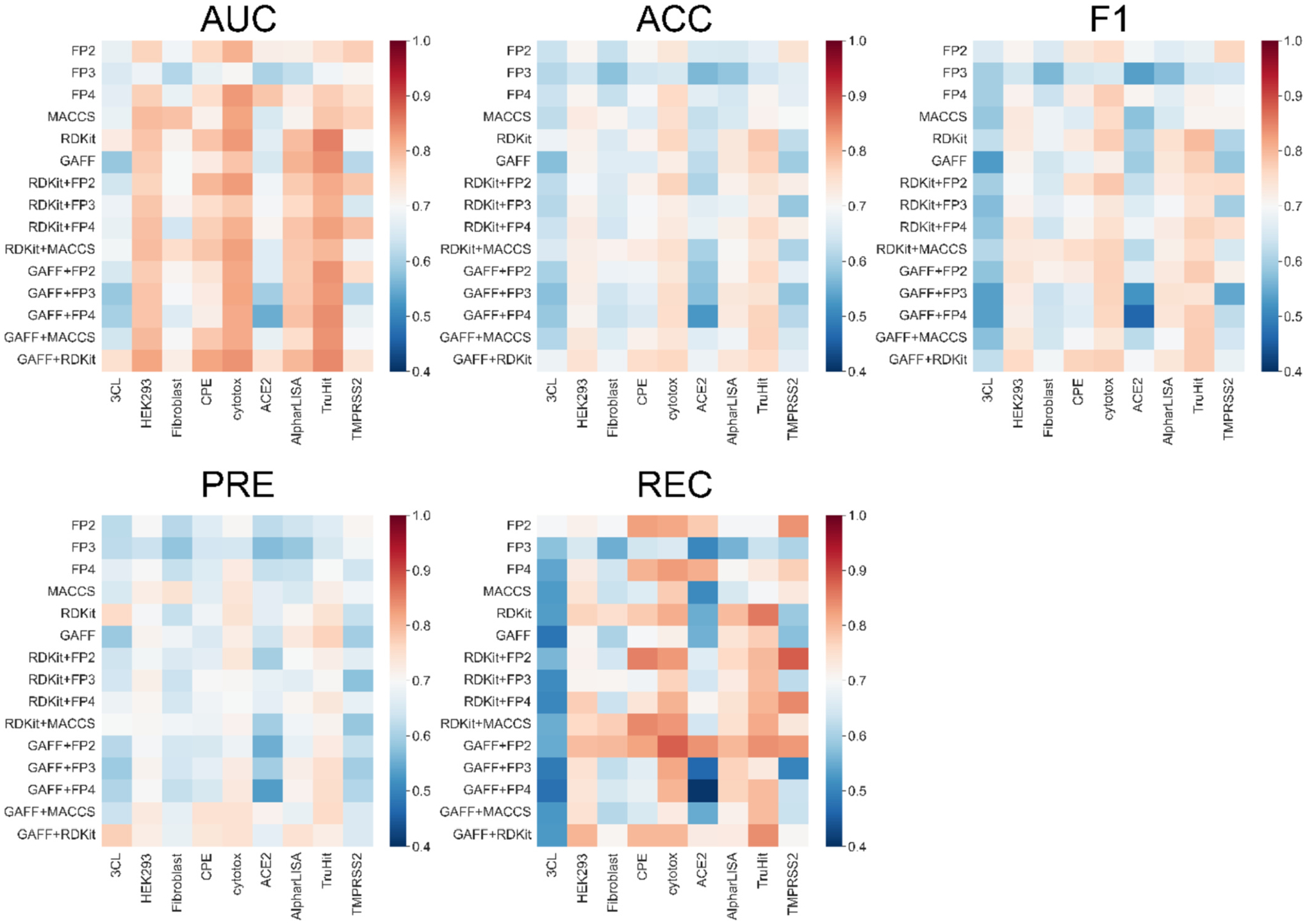
Heatmaps of metrics AUC, ACC, F1, PRE and REC for KNN models using different molecular descriptors.

**Fig. 4.**
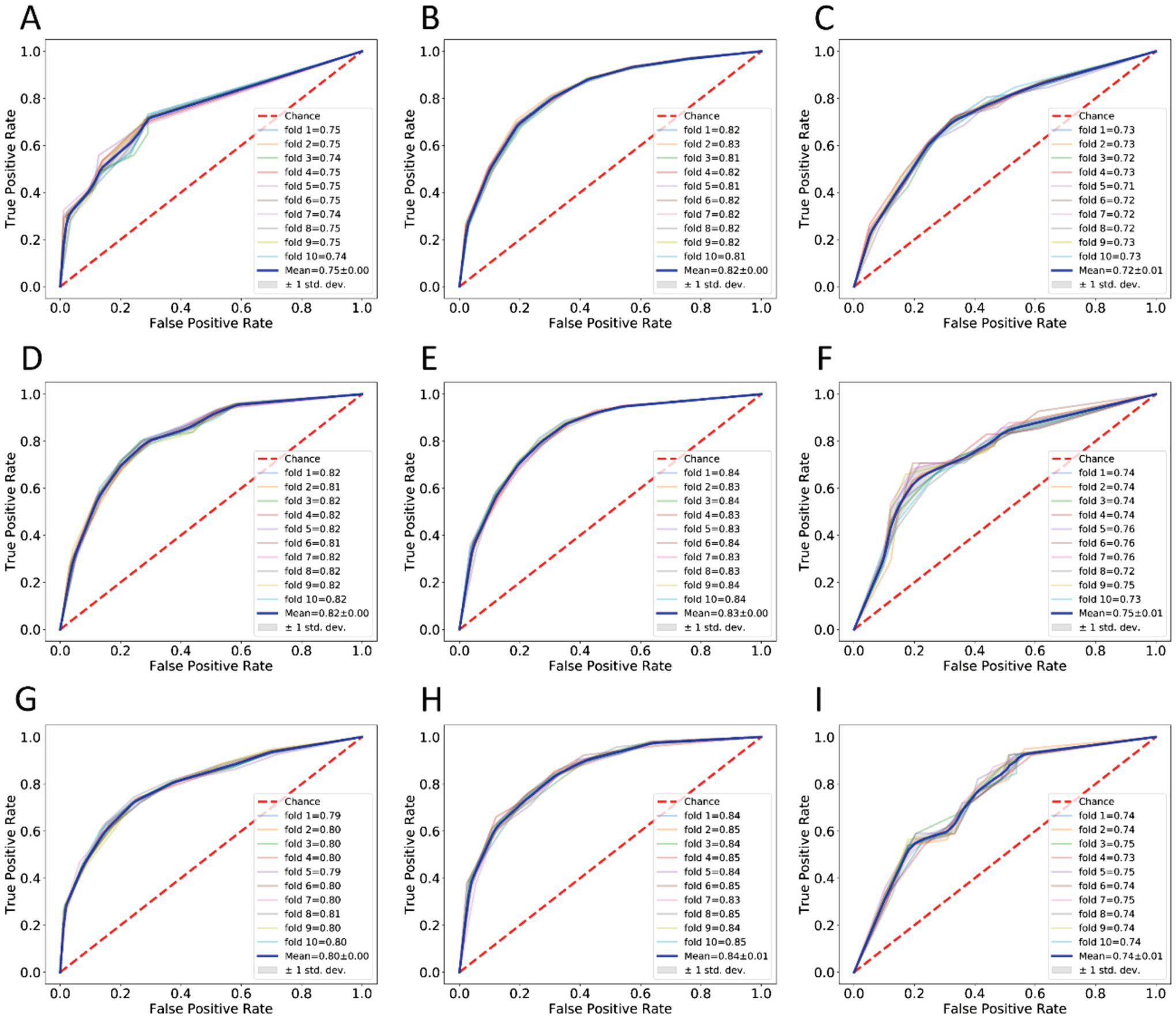
ROC curves of KNN models using GAFF+RDKIT as inputs. A-I are different assays. A. 3CL, B. HEK293, C. Fibroblast, D. CPE, E. Cytotox, F. ACE2, G. AlphaLISA, H. TruHit, I. TMPRSS2.

We compared the impact of different molecular descriptors on the model performance. For the sake of comparison, heatmaps which illustrate the values of a performance metric with colors were generated (Fig. 3). In these heatmaps, we used red to blue colors to indicate the large or small value of a performance metric, the higher the value, the more reddish color it is, and the lower the value, the more bluish color it is. The purpose of these visualizations is to provide a comprehensive overview of how well each descriptor performs across all assays and metrics. The heatmaps illustrate the overall performance of fifteen molecular descriptors applied for the construction of KNN models for 9 screening assays. All the 5 metrics should be considered to identify the best descriptor for all screening assays. By combining the results in both Fig. 3 and Fig. S3, overall, the GAFF+RDKit descriptor outperformed others since there were the least number of blue grids for it cross all the metrics and assays. Table 5 lists the average metrics scores of KNN models for each molecular descriptor in all assays. According to this table, GAFF+RDKit has comparable performance with other descriptors on the validation sets, albeit it has the highest AUC and PRE values. However, for the test sets, this descriptor achieves the best performance among all the descriptors as measured by all the performance metrics. Thus, GAFF+RDKit was selected as the default descriptor. GAFF, the abbreviation of General AMBER Force Field, is designed to describe subtle chemical environments using atom types [31]. GAFF was parametrized to be consistent with AMBER biomolecular force fields for studying protein-ligand and nucleic acid-ligand interactions. It can describe a wide range of organic or pharmaceutical molecules that are constituted of H, C, N, O, S, P, F, Cl, Br and I. Utilizing the companion software tool, Antechamber, GAFF atom type-based descriptor can be automatically generated for arbitrary organic molecules that can be modelled by GAFF [32]. Unlike fingerprint-based descriptors which only indicate the existing or non-existing of a certain substructure or structural pattern, GAFF descriptor encodes the total occurrences of subtle chemical environment in a molecule. On the other hand, RDKit is a popular open-source cheminformatics tool kit which can describe and collect molecule-level properties. GAFF+RDKit, combined by the features of both GAFF and RDKit, can better discriminate the actives from the inactives for all the screening assays than either of single type of descriptor. Fig. 4 illustrates the ROC curves of KNN models using GAFF+RDKIT descriptor for each assay. The ROC curves represent the performance of the KNN models in 10-fold cross-validation. It is notable that the AUC values of ROC curves for all 10 folds exhibit consistency, suggesting that our models have been effectively trained without over-fitting. Together with four other performance metrics (ACC, F1, PRE, REC) listed in Table 6, the performance for KNN models of GAFF+RDKit molecular descriptor for all assays can be comprehensively evaluated. As shown in Table 6, for validation set, the metrics scores of most metrics for the six assays are better than the scores in KC et al.’s study. As for the test set, overall, our models achieved better scores than KC et al.’s in performance metrics for four assays.

### 3.4. Evaluation of model predictivity using external test sets

To confirm the reliability of the constructed models, we additionally evaluated the performance of the models (COVID-19-CP, **P** in brief) in best scenarios (KNN algorithm and GAFF+RDKit fingerprint) on 4 different categories of external test sets compiled from different sources, which are: (1) the NCATS compounds not participating model construction, (2) the reported drugs/compounds that have been used or tested in COVID-19 treatment, (3) the reported compounds which are active in SARS-CoV-2-related bioassays, and (4) the screening compounds from ZINC database [33] (https://zinc.docking.org/) serving the negative control, i.e., those compounds are assumed as inactives. The model performance can be critically evaluated by using the five metrics (AUC, ACC, F1, PRE and REC) for the four external test sets.

#### 3.4.1. Test Set 1 – NCATS screening compounds

**Fig. 5.**
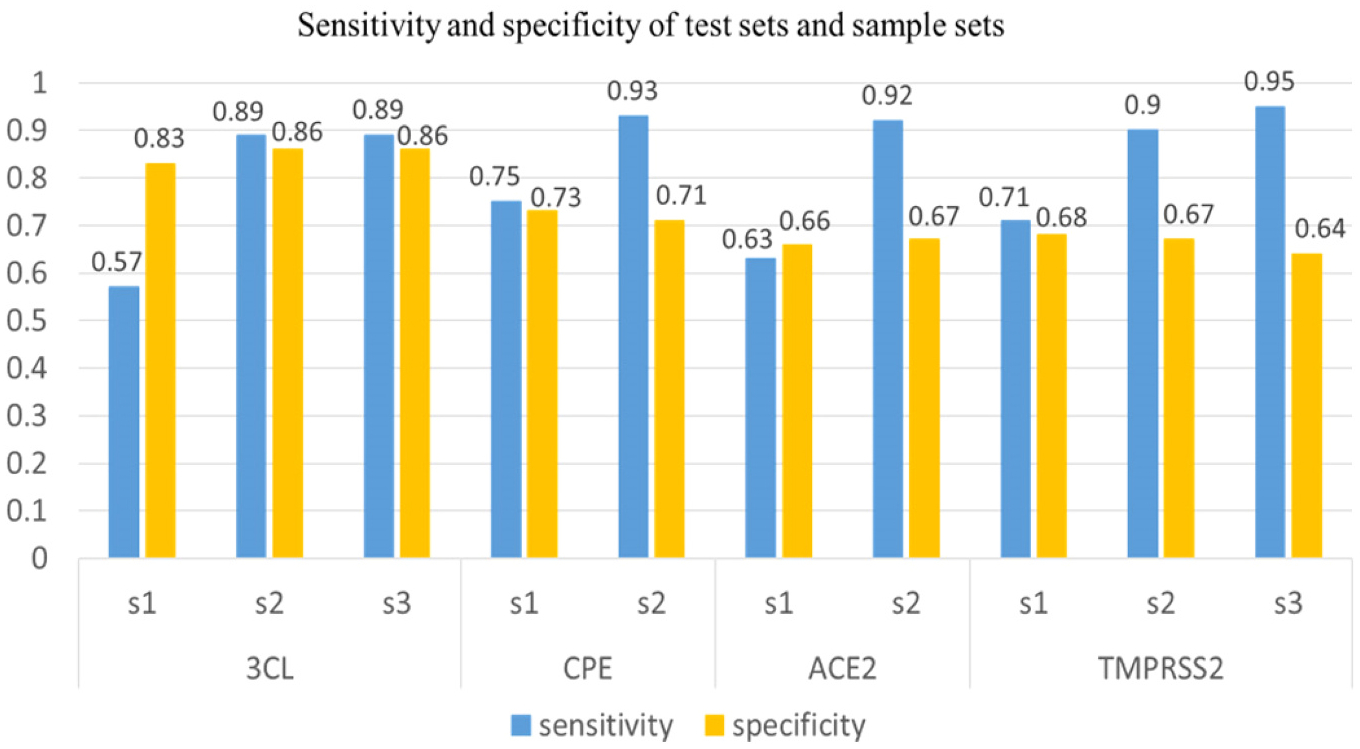
Sensitivity and specificity of test sets and sample sets in 3CL, CPE, ACE2, TMPRSS2 assays.

As described in the Data Set Preparation session, we have randomly divided inactive compounds for each of the four assays (3CL, CPE, ACE2 and TMPRSS2) into 2–4 subsets based on the actual number of inactives. While one inactive subset (s1) was randomly selected to participate ML-based model construction, we used inactive compounds from other sample sets (s2, s3, s4) to conduct external prediction. For the sake of computing the five metrics, we included the actives of each assay in the test sets. The predicted results of external datasets are displayed in Fig. 5. A striking feature of this figure was that the sensitivity scores of most assays were very high (>0.90), likely due to the participation of actives in model construction as well. The specificity scores of those external test sets, ranged from 0.60 to 0.86, were comparable to those reported for the test sets in model construction (Table 3). The similar specificity scores suggest that our models were not overfitted. Note that sensitivity measures the percentage of compounds predicted to be active out of the compounds which are active confirmed in bioassay, while specificity measures the percentage of the compounds predicted to be inactive out of the compounds which are inactives confirmed in bioassay [34]. Encouragingly, the specificity scores of external test sets were relatively high and comparable to the sensitivity scores, indicating that our models have the ability to rule out both the false positives and false negatives at the same time.

#### 3.4.2. Test Set 2: known anti-SARS-CoV-2 drugs in multiple assays

To validate the applicability of our models, we collected 28 compounds [35,36] that have been used or tested in COVID-19 treatment. 22 out of 28 compounds are approved drugs. We predicted their activities in different assays using both “REDIAL-2020” by KC et al. and our models. Table S1 lists the prediction results for each assay by utilizing REDIAL-2020 (**R**) and our model, COVID-19-CP (**P**), as detailed below. For all 28 compounds, the screening activities reported by NCATS Covid-19 OpenData Portal served as references. For a compound, if the predicted activity, active or inactive, is the same as the measured one, the number of correct predictions increases one, otherwise zero. If the predictions for the six assays (3CL, CPE, Cytotox, ACE2, AlphaLISA, TruHit) are all correct, the number of correct predictions is 6. We calculated the number of correct predictions for each compound by using REDIAL-2020 (**R**) and our predictor COVID-19-CP (**P**). Overall, the prediction results of **P** were better than **R** in term of the number of correct predictions. 13 compounds obtained correct predictions by **P** that are more than those by **R**, while 8 compounds with fewer correct predictions, and the rest of 7 compounds had equal correct predictions. When the performance of a specific assay was concerned, the percentage of correct prediction differs between **R** and **P** from one assay to another. The percentages of correct prediction were similar for Cytotox (~60%) and TruHit (~40%); **P** had larger percentages of correction prediction for 3CL (75% vs 68%), CPE (82% vs 68%) and ACE2 (61% vs 50%), while **R** achieved a better performance for AlphaLISA (71% vs 61%).

Two interesting compounds are Chloroquine and Hydroxy-chloroquine, which were hypothesized to be ACE2 blockers, however, the ACE2 assay suggest both two compounds are inactive. As shown in Table S1, for those two compounds, **P** made correct prediction, in contrast, **R** made the opposite prediction.

**Fig. 6.**
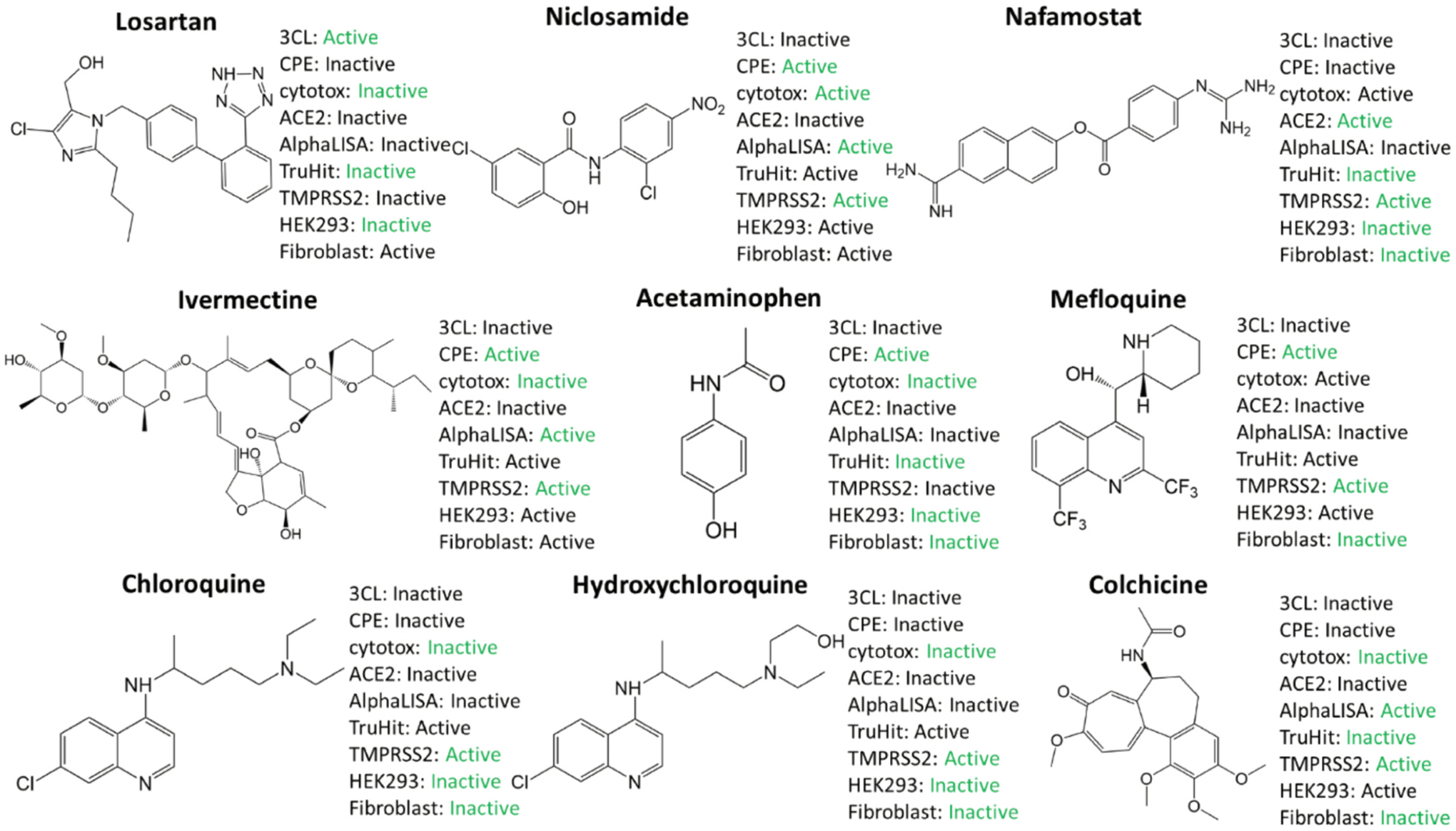
the structures of 9 potential candidates and the predictions of assays by P.

A set of 9 drug molecules predicted to have potential to be repurposed to treat for COVID-19 are shown in Fig. 6. Those drug molecules meet the following two criteria: 1. The predicted accuracy of **P** is higher than that of **R**; 2. The number of correct predictions by **P** is larger than 3, in other words, the overall prediction accuracy is higher than 50%. The assays correctly predicted by **P** were colored in green. It is shown that our predictor can correctly predict the activities of these drugs for most of assays. In addition, the developed models can predict activities of extra assays. For example, Nafamostat is the TMPRSS2 inhibitor [37], and its activity on TMPRSS2 assays was correctly predicted by our model.

#### 3.4.3. Test set 3: additional active compounds in individual bioassays

We further evaluated the performance of **P** using an external CPE dataset which was also adopted by KC et al. in their model evaluation process. Table S2 lists the names and SMILES of the 24 drugs which are actives in CPE assay. Among the 24 compounds, 19 of them were correctly predicted as active by our model, while only 5 of them were predicted as inactive. Thus, our model achieved a prediction accuracy of 79.2% for the external data set. In contrast, the percentage of correct prediction by **R** was 66.7%, obviously lower than our model. The prediction results by **R** and **P** for 21 3CL inhibitors collected from Kuzikov, et al. [38] were also compared. As shown in Table S3, the percentages of correct predictions by **P** (38.1%) are lower than **R** (61.9%). Thus, **P** achieved a comparable performance to **R** for the CPE external test set rather than the 3CL test set. To improve the prediction performance of **P** for the 3CL test set, we reconstructed the model using the screen data reported in Kuzikov, et al.’s study. The detail for the model reconstruction is described in the next section.

#### 3.4.4. Test set 4: screening compounds serving as negative control

The above three test sets mainly evaluated the models’ ability to identify true actives, while this test set can be applied to assess the models’ ability to reduce false positives. To this aim, we randomly collected 100 screening compounds from the ZINC database and assuming those compounds are inactive in the screenings. Table S4a–b lists the activities predicted by **R** and **P** for three assays (3CL, CPE, AlphaLISA) which directly measure a compound’s antiviral activities. The prediction results of 3CL, CPE and AlphaLISA assays by **R** and **P** were compared. According to Table S4, 33 out of 100 compounds had fewer positive predictions by **P** than **R**, while 31 compounds had fewer positive predictions by **R** than **P**. The results indicated that **P** performed slightly better than **R**. In hence, our developed model performs better than **R**, not only for the known inhibitors (positive control), but also for the screening compounds (negative control). Among the 100 screening compounds, 37 and 10 were predicted to be active for AlphaLISA assay and 3CL assay by **P**, respectively. It is of noted that the proportion of inactives in the screening set was close to the proportion of actives in the total dataset for both assays. Specifically, the active rate is 1018/(2269 +1018)= 31.0% for the AlphaLISA assay, while the value is 431/(431 +2916)= 12.9% for the 3CL assay. The similar positive rates of both assays demonstrated the high reliability of our models.

### 3.5. New 3CL model construction

Specially, to further enhance the model performance for 3CL assay, we constructed a second model for the 3CL protease using the screen data reported by Kuzikov, et al. to further improve the performance of **P**. The structures in SMILES and the inhibition data of screening compounds were first collected and 7662 compounds left after data cleaning. Then the compounds were ranked based on their percent inhibition values, and those with percent inhibition values larger than 25% were allocated into the active set, while the rest of compounds were randomly allocated into two inactive subsets. In detail, there are 342 compounds in active set, 3665 in s1 inactive subset, and 3655 compounds in s2 inactive subset. As we did for the NCATS 3CL assay, s1 subset was selected to construct and test the ML-based models, while s2 served as an external dataset for further model validation.

The treatment of data imbalance and model construction were the same as we did for the NCATS 3CL assay. Again, the KNN algorithm and RDKit+GAFF molecular descriptor was employed for model construction. Table 7 shows the scores of performance metrics for the new 3CL model. According to the table, for both validation and test sets, every metric has better predicted score for the new 3CL model than that constructed using the NCATS data. The sensitivity and specificity scores of s1 were 0.62 and 0.89, which were better than the corresponding scores of NCATS 3CL s1 subset predicted by the old 3CL model (0.57 and 0.83). As for the external sets, the sensitivity and specificity scores of s2 by the new 3CL model were 0.93 and 0.89, respectively, both higher than the corresponding values achieved by the old 3CL model for the NCATS 3CL s2 (0.89 and 0.86) and s3 (0.89 and 0.86) subsets.

In addition to the evaluation of inactive subsets, we evaluated the new 3CL model for 3 test sets as we did for the 3CL model constructed using the NCATS 3CL assay. After the old 3CL model was replaced with the new 3CL model, a new set of models was formed. For the known anti-SARS-CoV-2 drugs test set, the overall performance did not change using the new **P**. There were still 13 compounds with correct predictions by **P** higher than those by REDAIL-2020, while 8 compounds were lower, and the rest of 7 compounds were equal. The prediction results by the new P for the drug molecules in 3CL assay were summarized in Table S5. The third test set is the active compounds in single 3CL assay. Since the compounds are originally from Kuzikov, et al.’s study, the performance of the new model for this set was expectedly better than the old model, with 16 out of 21 compounds predicted active against only 8 compounds predicted active by the old model (Table S6). The fourth test set is the 100 screening compounds which serve as the negative control. Compared to the positive effect brought by the new 3CL model, the overall performance of new **P** on the prediction of screening compounds slightly decreased. As shown in Table S7, 30 out of 100 compounds have fewer predictions as “active” by new **P** than **R**, which is less than the number of compounds (37) with fewer predictions as “active” by **R** than **P**. Overall, the second 3CL model can improve the performance of **P** on the positive control but negatively affect the performance of **P** on the negative control. Therefore, we may adopt the old or new 3CL models according to our research aims.

### 3.6. Importance of input features

We applied SHAP analysis to identify key structural features which make the largest contributions to the best KNN models using GAFF+RDKit descriptor. Considering we utilized an external CPE dataset for model evaluation and obtained a satisfying prediction result, CPE assay was used an example for interpreting the SHAP analysis result. The GAFF+RDKit descriptor is consisted of 47 GAFF features and 208 RDKit features. Specifically, GAFF atom types precisely depict chemical environments and those key features identified by Shapley analysis indicate those structural features are potential pharmacophores for a compound to manifest a certain bioactivity. The description of GAFF and RDKit features was listed in Tables S8 and S9. We analyzed the contribution of each feature for the external CPE test set using the KNN model.

**Fig. 7.**
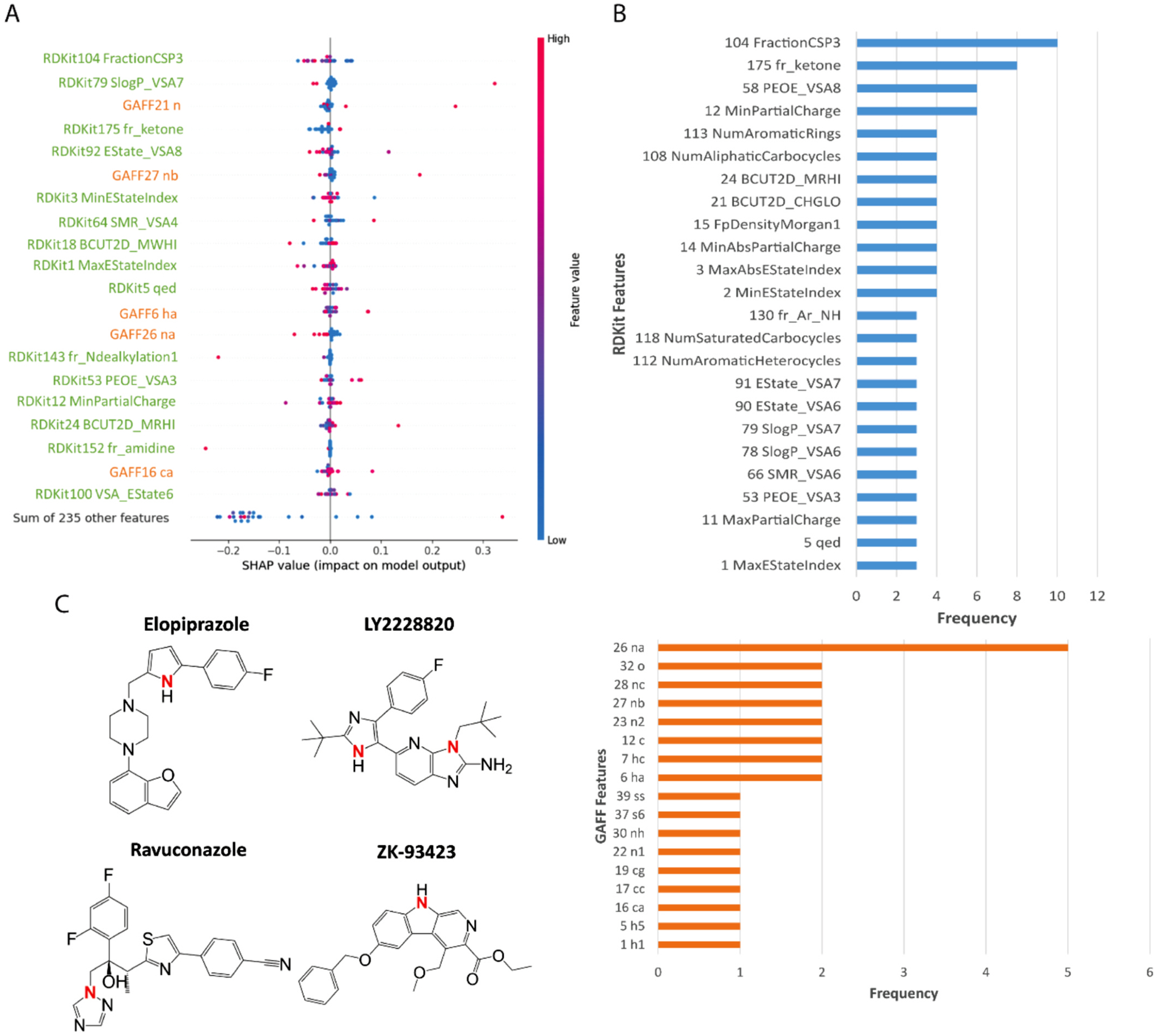
Importance of top input molecular descriptors. A. Importance of top 20 molecular descriptors and their SHAP values. B. Frequency distribution plots of RDKit features and GAFF features. C. An example of one important GAFF feature (GAFF 26: na).

In SHAP analysis, a positive SHAP value improves the prediction output and thus, the probability of a compound being active in the CPE assay, on the contrary, the negative value decreases the probability. To analyze the contribution of input features to the output of the prediction model, we made a summary plot for the top 20 input features and their SHAP values. In addition, top 9 most important features and their SHAP values for each compound with their chemical structures was depicted in waterfall plots (Figs. S4A–B). Fig. 7 generally shows the importance of top input molecular descriptors. Specifically, important features for each compound were summarized in Fig. 7A. In the plot, the actual value of each feature is represented by a color bar with all features in the GAFF and RDKit formats. The color transits from red to blue as the feature values decrease from high to low. Along the x-axis, we depicted the SHAP value, where a positive SHAP value increases the prediction output and therefore and therefore enhancing the probability that a compound aligns with the expected prediction (inactive for compounds in the counter-screen category and active in the other three categories). On the contrary, a negative SHAP value indicates a decrease in the probability. Each feature’s contribution to each molecule is visualized as a point on the graph. To be noted, when a feature exhibits a higher value (red) and a positive SHAP value, it increases the prediction output, making it favorable for achieving the anticipated activity in the assay. On the other hand, to improve the comprehensive assessment of the feature importance, we generated separate frequency distribution plots for GAFF and RDKit features, as depicted in Fig. 7B. These plots provide a clearer understanding of the relative importance and occurrence of those features in the relevant molecules.

Fig. 7A illustrated that *FractionCSP3* was the most important RDKit feature for CPE compounds. *FractionCSP3* is the fraction of C atoms that are sp^3^ hybridized. This finding was further validated in the frequency distribution plot in Fig. 7B, as this RDKit feature was important for 10 out 24 of compounds. By analyzing the structures of the testing compounds (Fig. S4), the presence of sp^3^ C atom was likely beneficial to increase the probability of a compound being active for CPE assay, since most of compounds contain this structural feature. The second important feature was RDKit feature *SlogP_VSA7*, which was related to the contribution of hydrophobic and lipophilic interactions to the overall solubility of a molecule in solvents. The importance of this feature indicated the high hydrophobicity on the molecular surface might have impact on the compound activity in CPE assay. The atom type of *n* in GAFF, which stands for the amide functional group, is recognized as an important feature. The GAFF feature *nb* was another important feature (Fig. 7A) and it represents aromatic nitrogen. The presence of this structural feature was considered to be beneficial to the CPE activity and 9 out of 24 compounds have aromatic nitrogen group. Furthermore, the histogram in Fig. 7B illustrated that GAFF feature *na* was also highly correlated to the CPE activity of a compound. *na* means the sp^2^ N with 3 substitutes. This feature has been considered to be very significant to 5 compounds according to the individual waterfall plots (Fig. S3) and has shown up in 4 of those compounds (Fig. 7C).

### 3.7. Dissemination of prediction models via COVID-19-CP web portal

To facilitate the dissemination of the prediction models, we developed a Web portal (https://clickff.org/amberweb/covid-19-cp). Users can access the web server that is integrated with the optimal KNN models and GAFF+RDKit molecular features for fast screening compounds that have potential treatment for COVID-19. Specifically, a user can open the webpage from a web browser, then input a molecular structure via different methods, and then submit the job to obtain the predicted activities of all 9 assays. Users can not only upload a mol2 or sdf file, but also draw 2-dimentional structures of compounds with a molecular Editor. Once the web portal receives the molecular structure (mol2/sdf/smi format), it will automatically generate GAFF+RDKit descriptors and feed the input data to the trained KNN models. After processing for a short time, the built-in models will provide the predicted activities of the input compound in 9 screening assays, which are 3 CL, HEK293, Fibroblast, CPE, Cytotox, ACE2, AlphaLISA, TruHit, TMPRSS2. Fig. S5 shows a sample submission page and the output page of the web portal. To summarize, an ideal anti-SARS-CoV-2 compound candidate meets the following criteria: (i) active in 3CL assay, (ii) inactive in HEK293 assay, (iii) inactive in Fibroblast assay, (iv) active in CPE assay, (v) inactive in Cytotox assay, (vi) active in ACE2 assay, (vii) active in AlphaLISA assay, (viii) inactive in TruHit assay, (ix) active in TMPRSS2 assay.

As shown in Fig. S4, in the output page, a structure similarity search section is provided immediate after the table summarizing the prediction result for users to search similar compounds in three databases, Drugbank [39,40], ChEMBL [41] and ZINC [33]. The defaulted cutoff value for similarity search is 0.8, indicating compounds with similarity equal to or higher than 0.8 compared to the query molecule found in the database will be outputted. However, if the applied cutoff does not lead to any hit, the most similar compound will be outputted. Users then can adjust the cutoff based on the Tanimoto coefficient of the most similar compound.

### 3.8. Attentive FP

The same data for the development of descriptor-based **P** models was adopted for the construction of Attentive FP (**A**) models. To completely integrate the published **A** models, instead of using SMOTE package to balance the data, we randomly divided compounds in inactive set into several subsets to make the number of inactives in each subset close to that of actives in the active set. The numbers of subsamples varied from one assay to another, depending on the ratio of actives and inactives in a specific assay. Accordingly, the same numbers of models were developed for each assay, and the average prediction results of validation and test sets were calculated and shown in Table 8. The values of evaluation metrics for each set were shown in Table S10.

Overall, the performance of **A** models on test sets was comparable to **P** models, and the average metrics values for 9 assays of A models, AUC= 0.77, ACC= 0.70, F1 = 0.70, PRE= 0.72, REC= 0.69, are close to the corresponding values of the P models, which are 0.79, 0.72, 0.72, 0.72, 0.74, respectively. For example, AUC, ACC, F1, PRE values on **A** model for 3CL assay were slightly lower than those on **P** model, but AUC, ACC, F1, PRE, REC values for AlphaLISA assay were higher than ones of the descriptor-based model.

Furthermore, the constructed **A** models were evaluated utilizing the same external test sets 2–4: known anti-SARS-CoV-2 drugs, compounds from 3CL and 3PE individual assays, screening compounds. The predicted activities were shown in Tables S1–S4. For the drug test set, again, we calculated the number of predictions that were consistent with experimental measurements for each compound. Among 28 drugs, 17 compounds had more correct activities predicted than **R**, the KC et al. models, while 5 compounds were lower. Compared to **P**, four more compounds had more correct predictions, indicating the prediction accuracy of test set 2 by **A** was slightly higher than **P**. For additional individual bioassays, Attentive FP achieved performance comparable to **P** and **R**. The same number of compounds was predicted to be active for 3CL assay by **A** compared to **R** (Table S3), while 14 compounds were predicted to be active for CPE assay (Table S2). As for the test set 4, **A** has slightly better performance than **P** and much better performance than **R**, as 30 out of 100 compounds had fewer positive predictions than **R** and only 15 out of 100 compounds had more positive predictions than **R**.

### 3.9. Consensus Prediction

**Fig. 8.**
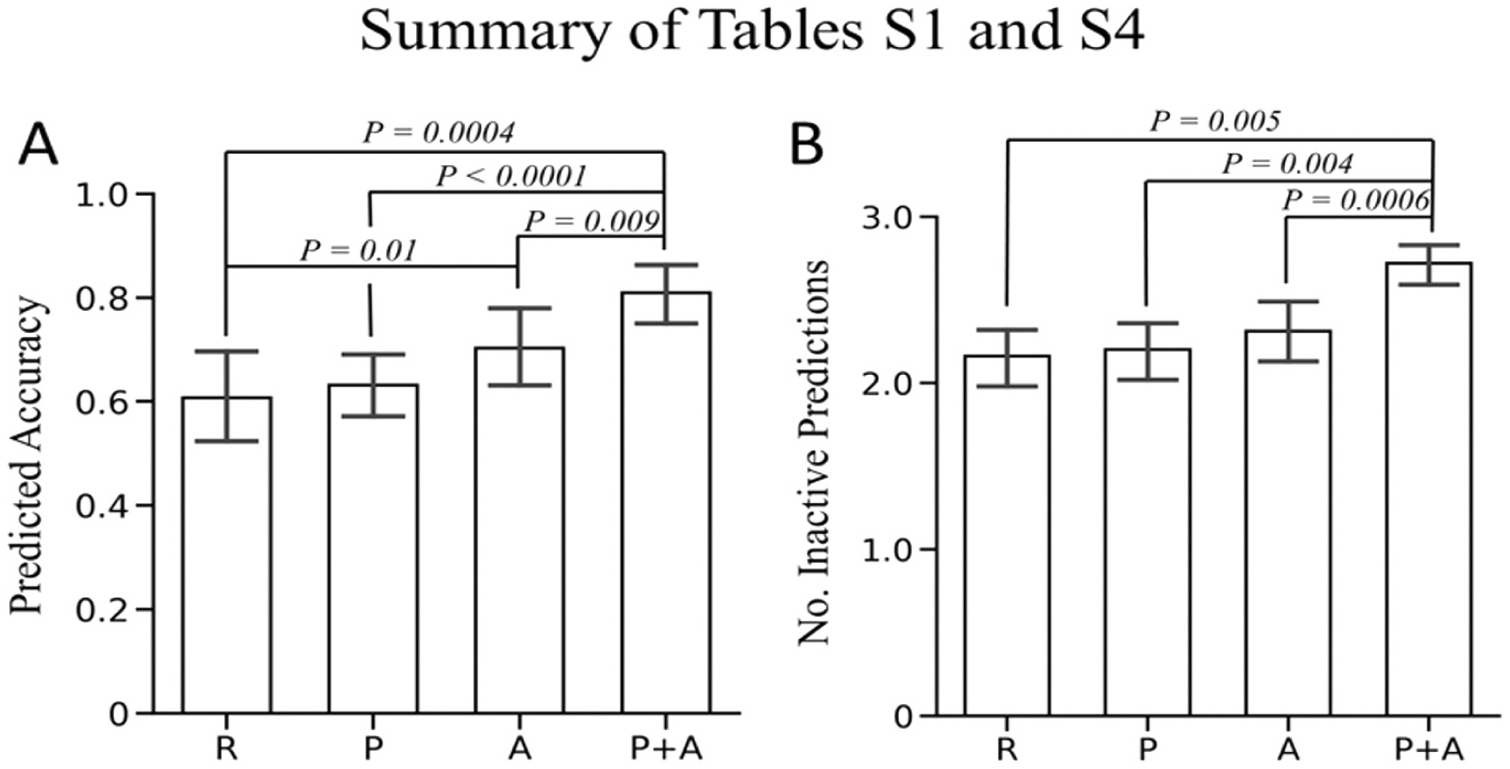
The prediction results among R, P, A, P + A models summarized from Tables S1 and S4. A. Table S1. B. Table S4.

**Fig. 9.**
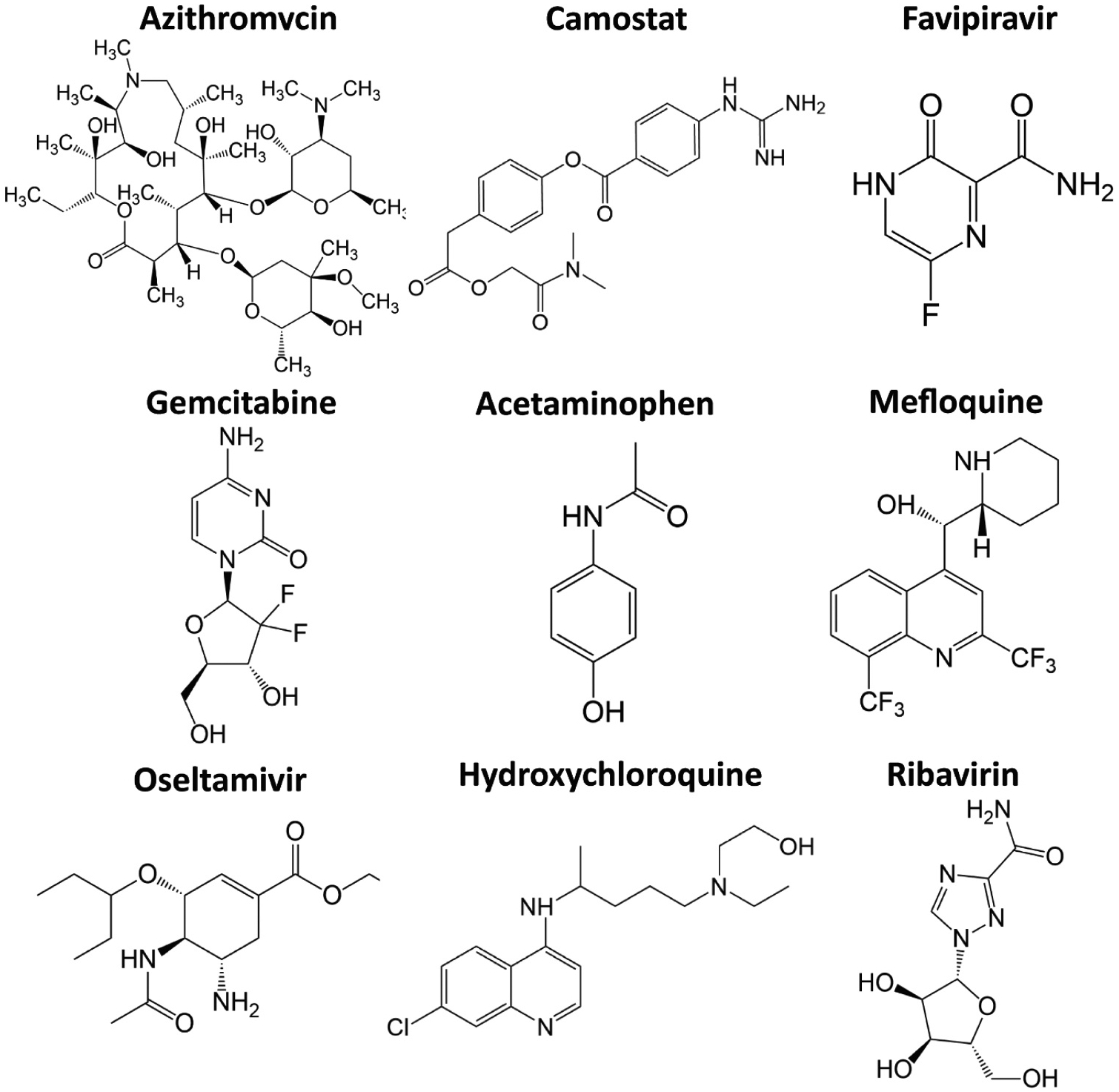
the structures of 9 drugs that were correctly predicted for all 6 assays by the consensus method.

To achieve the better performance, we analyzed the consensus scores by combining the prediction results from **P** and **A**. For any compound, the predicted activity using the consensus method (**P + A**) follows the rules below: (1) if it is predicted to be active by both **P** and **A** for an assay, it is considered to be active (2) if it is predicted to be active by either **P** or **A**, it is considered to be inactive (3) if it is predicted to be inactive by **P** and **A**, it is considered to be inactive. The performance of consensus model was evaluated using test sets 2 and 4. For the drug test set, comparing to **R**, there were 20 compounds having more correct predictions than R, while only 2 compounds have fewer correct predictions than **R**, and the numbers of correct prediction are equal for **P + A** and **R** for the rest of 6 compounds (Table S1). Among 28 drugs in the dataset, there were 9 of them were predicted exactly consistent with the experimental activities in all 6 assays (3CL, CPE, ACE2, AlphaLISA, TruHit, Cytotox) using the consensus method, and their chemical structures were shown in Figs. 8 and 9. As for the screening test set, as shown in Table S4, half of the compounds were predicted to have fewer positive activities using the **P + A** and among rest of them. Only 10% of compounds were predicted to have more positive predictions. Additionally, we performed Wilcoxon signed-rank test [42], to compare the prediction accuracy between two models for external test set 2 (Table S1) and external test 4 (Table S4). The overall comparison results as well as the statistical p values were shown in Fig. 8. It is obvious that the consensus model, P + A, significantly outperformed other three individual models for both external test sets. Therefore, the consensus method which mixes the prediction results from both descriptor-based and graph-based models indeed effectively improved the prediction performance and outperformed all other developed models in this work.

## 4. Conclusions

We introduced a series of predictive models to accurately predict the anti-SARS-CoV-2 activities of screening compounds. The impact of 6 different ML algorithms in combination with 15 molecular descriptors for 9 screening assays belonging to four categories on the models was deeply explored. We found that the developed predictive models utilizing the KNN method using the molecular descriptor, GAFF+RDKit, achieved the best overall performance for all nine assays. Among the 4 common performance metrics (AUC, ACC, F1, PRE), our optimal prediction models achieved much better predicted scores for 6 assays than those proposed in KC et al.’s study. We have extensively evaluated the predictive models using four external test sets including a negative control test set consisting of 100 druglike screening compounds from ZINC database. The second 3CL model utilizing the screen data from Kuzikov, et al.’s study has improved the performance of positive prediction, but decreased the performance of negative prediction as well, suggesting there is a trade-off on different performance metrics for a given model. SHAP analysis results indicated that sp^3^ C atom, sp^2^ N atom and aromatic N were important to a compound being active in CPE assay. In addition, the hydrophobicity may also have an impact on the compound activity in CPE assay. We have developed a webtool, COVID-19-CP, allowing users to predict a compound’s anti-SARS-CoV-2 activities using virtual input formats, and searching similar compounds from three mainstream databases. **P** is available on GitHub (https://github.com/Mayjig/COVID-19-CP_batch_screen) as well for the batch screening of potential anti-SARS-CoV-2 compounds. Furthermore, Attentive FP (**A**) was used in this study as an example of graph-based model to be compared with the traditional descriptor-based model (**P**). We used the same external test sets to evaluate the performance of **A**. As a result, the performance of **A** was comparable to **P**, and both of them outperformed **R**. The consensus model was derived by combining the **A** and **P**, and its performance was way much better than all **A**, **P** and **R**. As such, the consensus scores of multiple models, especially those were constructed using different descriptors and machine learning algorithms, can effectively improve the prediction accuracy.

## Supplementary Material

1

## Figures and Tables

**Table 1:** Summary of counts for datasets.

Assay		Total		[(Training (90%) & Validation (10%)] (80%)		Test (20%)	
Assay Abbr.^a^	Category	Actives	Inactives	Actives	Inactives	Actives	Inactives
3CL	1	431	2916	343	2826	86	86
CPE	2	841	4808	648	4623	168	168
ACE2	3	203	1574	162	1533	41	41
AlphaLISA	3	1018	2269	812	2060	204	204
TMPRSS2	3	194	1597	155	1558	39	39
Cytotox	4	1685	7844	1325	7494	337	337
TruHit	4	1030	2257	819	2045	206	206
HEK293	4	4307	5303	3376	4395	861	861
Fibroblast	4	590	4004	467	3868	118	118

a 3CL: 3CL enzymatic activity; CPE: SARS-CoV-2 cytopathic effect CPE; ACE2: ACE2 enzymatic activity assay; AlphaLisa: Spike-ACE2 protein-protein interaction AlphaLISA assay; TMPRSS2: TMPRSS2 enzymatic activity assay; Cytotox: SARS-CoV-2 cytopathic effect counter-screen assay; TruHit: Spike-ACE2 protein-protein interaction TruHit conunterscreen assay; HEK293: HEK 293 cell line toxicity assay; Fibroblast: human fibroblast toxicity assay.

**Table 2:** Average scores of metrics for models with six ML algorithms of all molecular descriptors in nine assays.

Datasets	Metrics	SVM	LR	DT	RF	KNN	CNB
Validation	AUC	0.88	0.88	0.81	0.87	0.91	0.77
	ACC	0.82	0.82	0.75	0.79	0.80	0.71
	F1	0.82	0.83	0.76	0.79	0.82	0.72
	PRE	0.81	0.83	0.74	0.77	0.73	0.69
	REC	0.84	0.83	0.80	0.82	0.94	0.76
Test	AUC	0.73	0.75	0.67	0.74	0.74	0.69
	ACC	0.66	0.66	0.63	0.67	0.68	0.64
	F1	0.59	0.58	0.62	0.65	0.69	0.64
	PRE	0.74	0.76	0.64	0.69	0.67	0.64
	REC	0.52	0.51	0.63	0.63	0.71	0.65

**Table 3:** Average scores of metrics for KNN models using all 15 molecular descriptors for each assay. The values highlighted in blue and bold font indicating the reported values are higher than those for the best models predicted by KC et al. Noted that TMPRSS2, HEK293 and Fibroblast were not studied by KC et al.

Datasets	Metrics	3CL	CPE	ACE2	AlphaLISA	TMPRSS2	Cytotox	TruHit	HEK293	Fibroblast
Validation	AUC	**0.93**	**0.93**	**0.93**	**0.87**	0.94	**0.93**	**0.89**	0.81	0.93
	ACC	**0.84**	**0.82**	**0.80**	0.78	0.80	**0.83**	0.80	0.74	0.82
	F1	**0.86**	**0.84**	**0.83**	0.80	0.83	**0.85**	0.82	0.75	0.84
	PRE	**0.77**	**0.74**	0.73	0.73	0.71	0.76	0.75	0.72	0.74
	REC	**0.97**	**0.98**	**0.98**	**0.89**	0.99	**0.97**	**0.90**	0.79	0.97
Test	AUC	0.66	**0.75**	0.67	0.76	0.71	**0.81**	**0.81**	0.78	0.70
	ACC	0.62	**0.69**	0.62	0.69	0.65	**0.74**	**0.74**	0.71	0.66
	F1	0.59	**0.71**	0.62	0.70	0.67	**0.75**	**0.74**	0.72	0.66
	PRE	0.65	**0.68**	0.63	0.68	0.64	**0.72**	0.72	0.70	0.65
	REC	0.54	**0.74**	0.62	0.73	0.70	**0.79**	**0.77**	0.74	0.67

**Table 4:** The average SRD results (%) for six ML algorithms across all assays and descriptors.

	SVM	LR	DT	RF	KNN	CNB
Validation	5.44	5.50	9.09	5.04	6.50	15.15
Test	36.25	36.03	38.92	35.75	34.61	38.83
Average	20.85	20.77	24.01	20.40	20.56	26.99

**Table 5:** Average scores of metrics for KNN models with 15 molecular descriptors in nine assays.

Datasets	Validation					Test				
Metrics	AUC	ACC	F1	PRE	REC	AUC	ACC	F1	PRE	REC
FP2	0.89	0.78	0.81	0.71	0.94	0.73	0.68	0.70	0.65	0.75
FP3	0.77	0.70	0.72	0.68	0.77	0.66	0.62	0.60	0.62	0.59
FP4	0.89	0.78	0.81	0.72	0.94	0.75	0.68	0.70	0.66	0.74
MACCS	0.92	0.82	0.84	0.76	0.94	0.75	0.69	0.68	0.70	0.67
RDKit	0.93	0.83	0.85	0.76	0.96	0.76	0.70	0.71	0.69	0.73
GAFF	0.92	0.82	0.84	0.76	0.94	0.74	0.68	0.68	0.69	0.66
RDKit+FP2	0.91	0.81	0.83	0.74	0.95	0.76	0.69	0.71	0.67	0.77
RDKit+FP3	0.92	0.84	0.84	0.76	0.94	0.74	0.68	0.68	0.67	0.69
RDKit+FP4	0.92	0.83	0.84	0.75	0.95	0.76	0.70	0.71	0.68	0.74
RDKit+MACCS	0.92	0.80	0.83	0.73	0.96	0.76	0.69	0.71	0.67	0.77
GAFF+FP2	0.91	0.79	0.81	0.70	0.96	0.74	0.68	0.72	0.65	0.81
GAFF+FP3	0.92	0.82	0.84	0.75	0.95	0.73	0.67	0.66	0.67	0.66
GAFF+FP4	0.92	0.81	0.84	0.75	0.95	0.73	0.67	0.67	0.67	0.68
GAFF+MACCS	0.93	0.83	0.85	0.76	0.96	0.75	0.70	0.68	0.71	0.67
GAFF+RDKit	0.93	0.83	0.84	0.76	0.95	0.79	0.72	0.73	0.71	0.75

**Table 6:** Scores of metrics for KNN model using GAFF+RDKit molecular descriptor for each assay. The values highlighted in blue and bold font indicating the reported values are higher than those for the best models predicted by KC et al. Noted that TMPRSS2, HEK293 and Fibroblast were not studied by KC et al.

Datasets	Metrics	3CL	CPE	ACE2	AlphaLISA	TMPRSS2	Cytotox	TruHit	HEK293	Fibroblast
Validation	AUC	**0.94**	**0.95**	**0.95**	**0.90**	0.95	**0.95**	**0.92**	0.84	0.94
	ACC	**0.86**	**0.84**	**0.83**	0.81	0.82	**0.85**	**0.83**	0.76	0.83
	F1	**0.88**	**0.86**	**0.85**	0.82	0.85	**0.87**	**0.84**	0.77	0.85
	PRE	**0.80**	**0.76**	**0.75**	0.76	0.74	**0.78**	0.78	0.74	0.75
	REC	**0.98**	**0.99**	**0.99**	**0.90**	1.00	**0.99**	**0.93**	0.80	0.99
Test	AUC	**0.75**	**0.82**	0.75	**0.80**	0.74	**0.83**	**0.84**	0.82	0.72
	ACC	0.68	**0.76**	0.68	0.74	0.67	**0.76**	**0.76**	0.75	0.69
	F1	0.63	**0.77**	0.70	0.74	0.68	**0.77**	**0.78**	0.76	0.69
	PRE	**0.77**	**0.74**	0.67	0.75	0.66	**0.75**	0.72	0.72	0.68
	REC	0.53	**0.80**	0.72	0.73	0.70	**0.80**	**0.84**	0.80	0.71

**Table 7:** Score of metrics for KNN model using GAFF+RDKit molecular descriptor for the second 3CL model.

Datasets	AUC	ACC	F1	PRE	REC
Validation	0.98	0.95	0.95	0.91	1.00
Test	0.77	0.76	0.72	0.85	0.62

**Table 8:** Scores of metrics for Attentive FP models for each assay.

Datasets	Metrics	3CL	CPE	ACE2	AlphaLISA	TMPRSS2	Cytotox	TruHit	HEK293	Fibroblast
Validation	AUC	0.77	0.77	0.78	0.80	0.74	0.83	0.90	0.84	0.82
	ACC	0.68	0.70	0.66	0.71	0.68	0.74	0.80	0.76	0.75
	F1	0.62	0.67	0.71	0.71	0.71	0.71	0.75	0.71	0.79
	PRE	0.58	0.69	0.84	0.68	0.65	0.77	0.76	0.73	0.79
	REC	0.68	0.67	0.65	0.76	0.79	0.67	0.76	0.69	0.80
Test	AUC	0.65	0.78	0.65	0.86	0.70	0.82	0.88	0.82	0.73
	ACC	0.62	0.72	0.62	0.77	0.63	0.73	0.77	0.75	0.68
	F1	0.62	0.71	0.59	0.78	0.67	0.71	0.76	0.73	0.69
	PRE	0.63	0.74	0.68	0.76	0.61	0.78	0.82	0.78	0.67
	REC	0.62	0.70	0.58	0.82	0.74	0.66	0.72	0.68	0.72

## Data Availability

The datasets were collected from the following links for each assay. 3CL: https://opendata.ncats.nih.gov/covid19/assay?aid=9. CPE: https://opendata.ncats.nih.gov/covid19/assay?aid=14. ACE2: https://opendata.ncats.nih.gov/covid19/assay?aid=6. AlphaLISA: https://opendata.ncats.nih.gov/covid19/assay?aid=1. TMPRSS2: https://opendata.ncats.nih.gov/covid19/assay?aid=8. Cytotox: https://opendata.ncats.nih.gov/covid19/assay?aid=15. TruHit: https://opendata.ncats.nih.gov/covid19/assay?aid=2. HEK293: https://opendata.ncats.nih.gov/covid19/assay?aid=20. Fibroblast: https://opendata.ncats.nih.gov/covid19/assay?aid=21. All code was implemented in Python using Keras as the primary machine learning package. The data, code and scripts are available at https://github.com/Mayjig/COVID-19-CP_batch_screen.
