# Supplementary material for: Predicting anti-SARS-CoV-2 activities of chemical compounds using machine learning models
Source: Artif Intell Chem. Author manuscript; Available in PMC 2023 Dec 12. (PMC10715801; doi:10.1016/j.aichem.2023.100029)
Supplement: 1 [file NIHMS1949310-supplement-1.docx]

**Supplemental Information**

**Development of Machine Learning Models to Predict a Chemical’s Anti-SARS-CoV-2 Activities**

Beihong Ji, Yuhui Wu, Elena N Thomas, Jocelyn N Edwards, Xibing He, Junmei Wang*

Department of Pharmaceutical Sciences and Computational Chemical Genomics Screening Center, School of Pharmacy, University of Pittsburgh, Pittsburgh, PA 15261, USA.

* Corresponding author, [junmei.wang@pitt.edu](mailto:junmei.wang@pitt.edu)

**Supplemental Text**

**Machine Learning Algorithms**

1. support vector machine (SVM): Support-vector machines are [supervised learning](https://en.wikipedia.org/wiki/Supervised_learning) models with associated learning [algorithms](https://en.wikipedia.org/wiki/Algorithm) that analyze data for [classification](https://en.wikipedia.org/wiki/Statistical_classification) and [regression analysis](https://en.wikipedia.org/wiki/Regression_analysis). The objective of the support vector machine algorithm is to find a hyperplane in an N-dimensional space (N: the number of features) that distinctly classifies the data points.

*svm.SVC(random_state=0, kernel='linear', probability=True)*

1. logistic regression (LR): Logistic regression is a process of modeling the probability of a discrete outcome given an input variable. The most common [logistic regression models](https://www.sciencedirect.com/topics/computer-science/logistic-regression-model) a binary outcome; something that can take two values such as true/false, yes/no, and so on.

*LogisticRegression(random_state=0, solver='liblinear')*

1. decision tree (DT): A decision tree is a flowchart-like structure in which each internal node represents a "test" on an attribute (e.g. whether a coin flip comes up heads or tails), each branch represents the outcome of the test, and each leaf node represents a class label (decision taken after computing all attributes). The paths from root to leaf represent classification rules.

*DecisionTreeClassifier(random_state=0, max_depth=5)*

1. Random Forest (RF): Random forests or random decision forests are an ensemble learning method for classification, regression and other tasks that operates by constructing a multitude of decision trees at training time. For classification tasks, the output of the random forest is the class selected by most trees.

*RandomForestClassifier(random_state=0, max_depth=5, n_estimators = 100)*

1. k-nearest neighbors (KNN): The KNN algorithm assumes that similar things exist in close proximity. In other words, similar things are near to each other. The k-nearest neighbors (KNN) algorithm is a data classification method for estimating the likelihood that a data point will become a member of one group or another based on what group the data points nearest to it belong to.

*KNeighborsClassifier(n_neighbors=7)*

1. complement Naïve Bayes (NB): Naive Bayes methods are a set of supervised learning algorithms based on applying Bayes’ theorem with the “naive” assumption of conditional independence between every pair of features given the value of the class variable. Multinomial Naïve Bayes implements the naive Bayes algorithm for multinomially distributed data, and is one of the two classic naive Bayes variants used in text classification The Complement Naive Bayes classifier was designed to correct the “severe assumptions” made by the standard Multinomial Naive Bayes classifier. It is particularly suited for imbalanced data sets.

*ComplementNB(alpha=1.0, force_alpha='warn', fit_prior=True, class_prior=None, norm=False)*

**Model Performance Metrics**

1. True positive (TP)

Correctly predicted positive values. The value of actual class is true and the value of predicted class is also true.

1. True negative (TN)

Correctly predicted negative values. The value of actual class is false and the value of predicted class is also false.

1. False positive (FP)

Incorrectly predicted negative values. The value of actual class is false and the value of predicted class is true.

1. False negative (FN)

Incorrectly predicted positive values. The value of actual class is true and the value of predicted class is false.

1. Accuracy

$$Accuracy= \frac{TP+TN}{TP+TN+FP+FN}$$

2. Precision

$$Precision= \frac{TP}{TP+FP}$$

1. Recall:

$$Recall= \frac{TP}{TP+FN}$$

2. F1-score:

$$F1-score=({\frac{{Precision}^{-1}+{Recall}^{-1}}{2})}^{-1}=2\times\frac{Precision\times Recall}{Precision+Recall}$$

3. Sensitivity

$$Sensitivity= \frac{TP}{TP+FN}$$

4. Specificity

$$Specificity= \frac{TN}{TN+FP}$$

**Table S1.** The comparison of prediction results for potential compounds among four models. “1” refers to active, while “0” refers to inactive. Green refers to the desirable activity, while red refers to the undesirable activity. “R”, “P”, “A”, “P+A” and “E” represent REDIAL-2020, the developed predictor COVID-19-CP, Attentive FP model, consensus model and experimental activity obtained from NCATS library, respectively.

**Table S2.** The comparison of prediction results for CPE dataset among four models. “R”, “P” and “A” represent REDIAL, the developed predictor COVID-19-CP and Attentive FP model, respectively.

| Drug | R | P | A |
| --- | --- | --- | --- |
| Droloxifene | 1 | 1 | 1 |
| Isoosajin | 1 | 1 | 1 |
| Isopomiferin | 1 | 1 | 1 |
| Osajin | 0 | 1 | 1 |
| Oxyclozanide | 1 | 1 | 0 |
| Salinomycin | 0 | 1 | 1 |
| Elopiprazole | 1 | 1 | 1 |
| ONO 5334 | 0 | 0 | 0 |
| SDZ-62-434 | 1 | 1 | 1 |
| SL-11128 | 1 | 0 | 0 |
| VBY-825 | 0 | 1 | 0 |
| Alisporivir | 0 | 1 | 0 |
| Avasimibe | 1 | 1 | 1 |
| Avatrombopag | 1 | 1 | 1 |
| Drotaverine | 1 | 1 | 1 |
| IPAG | 1 | 1 | 1 |
| JTE-013 | 0 | 1 | 0 |
| LY2228820 | 1 | 0 | 0 |
| Mibampator | 1 | 0 | 0 |
| Pevonedistat | 0 | 1 | 0 |
| Polidocanol | 0 | 0 | 0 |
| Ravuconazole | 1 | 1 | 1 |
| VLX600 | 1 | 1 | 1 |
| ZK-93423 | 1 | 1 | 1 |
| Total | 16 | 19 | 14 |

**Table S3.** The comparison of prediction results for 3CL dataset among three models. “R”, “P” and “A” represent REDIAL, the developed predictor COVID-19-CP and Attentive FP model, respectively.

| Drug | R | P | A |
| --- | --- | --- | --- |
| Baicalein | 1 | 1 | 1 |
| Benserazide Hydrochloride | 1 | 1 | 0 |
| Bonaphthone | 1 | 0 | 1 |
| Bronopol | 1 | 1 | 0 |
| Carmofur | 0 | 0 | 1 |
| Dihydrexidine | 1 | 0 | 1 |
| Disulfiram | 1 | 0 | 1 |
| Felbinac Ethyl | 0 | 1 | 1 |
| MG-132 | 1 | 0 | 0 |
| ML311 | 0 | 0 | 0 |
| MLS0315771 | 0 | 0 | 1 |
| Myricetin | 1 | 0 | 1 |
| Oltipraz | 0 | 1 | 1 |
| PR-619 | 1 | 1 | 1 |
| Proflavine Hemisulfate | 1 | 0 | 1 |
| SKF-38393 | 1 | 1 | 1 |
| TDZD-8 | 0 | 0 | 0 |
| Thioguanosine | 0 | 0 | 0 |
| Tideglusib | 1 | 0 | 1 |
| WAY-308264 | 0 | 0 | 0 |
| Z-DEVD-FMK | 1 | 1 | 0 |
| Total | 13 | 8 | 13 |

**Table S4a.** The comparison of prediction results for randomly collected drugs among four models. “1” refers to active, while “0” refers to inactive. Green refers to the desirable activity, while red refers to the undesirable activity. “R”, “P”, “A” and “P+A” represent REDIAL-2020, the developed predictor COVID-19-CP, Attentive FP model and consensus model obtained from NCATS library, respectively.

| Compounds | 3CL | | | | CPE | | | | AlphaLISA | | | | No. Inactive Predictions | | | |
| --- | --- | --- | --- | --- | --- | --- | --- | --- | --- | --- | --- | --- | --- | --- | --- | --- |
|  | R | P | A | P+A | R | P | A | P+A | R | P | A | P+A | R | P | A | P+A |
| ZINC00927555 | 0 | 0 | 0 | 0 | 1 | 0 | 1 | 0 | 1 | 0 | 1 | 0 | 1 | 3 | 1 | 3 |
| ZINC01669393 | 1 | 0 | 1 | 0 | 1 | 1 | 1 | 1 | 1 | 1 | 1 | 1 | 0 | 1 | 0 | 1 |
| ZINC01835455 | 0 | 0 | 0 | 0 | 0 | 1 | 0 | 0 | 1 | 1 | 1 | 1 | 2 | 1 | 2 | 2 |
| ZINC02038278 | 1 | 0 | 0 | 0 | 0 | 0 | 0 | 0 | 0 | 0 | 0 | 0 | 2 | 3 | 3 | 3 |
| ZINC02245307 | 1 | 0 | 1 | 0 | 0 | 0 | 0 | 0 | 0 | 0 | 0 | 0 | 2 | 3 | 2 | 3 |
| ZINC03049271 | 1 | 0 | 1 | 0 | 0 | 0 | 0 | 0 | 1 | 0 | 1 | 0 | 1 | 3 | 1 | 3 |
| ZINC04503565 | 0 | 1 | 0 | 0 | 0 | 0 | 0 | 0 | 0 | 0 | 0 | 0 | 3 | 2 | 3 | 3 |
| ZINC05626166 | 0 | 0 | 1 | 0 | 0 | 0 | 0 | 0 | 0 | 1 | 0 | 0 | 3 | 2 | 2 | 3 |
| ZINC06198146 | 0 | 0 | 0 | 0 | 0 | 0 | 0 | 0 | 1 | 0 | 1 | 0 | 2 | 3 | 2 | 3 |
| ZINC06637259 | 0 | 0 | 0 | 0 | 0 | 0 | 0 | 0 | 0 | 1 | 0 | 0 | 3 | 2 | 3 | 3 |
| ZINC06805870 | 0 | 0 | 0 | 0 | 1 | 1 | 0 | 0 | 0 | 1 | 0 | 0 | 2 | 1 | 3 | 3 |
| ZINC08063873 | 0 | 0 | 0 | 0 | 1 | 1 | 1 | 1 | 1 | 0 | 1 | 0 | 1 | 2 | 1 | 2 |
| ZINC08849480 | 0 | 0 | 0 | 0 | 1 | 1 | 0 | 0 | 0 | 1 | 0 | 0 | 2 | 1 | 3 | 3 |
| ZINC08857316 | 1 | 1 | 1 | 1 | 1 | 1 | 1 | 1 | 1 | 1 | 1 | 1 | 0 | 0 | 0 | 0 |
| ZINC09241936 | 0 | 0 | 1 | 0 | 1 | 1 | 1 | 1 | 1 | 1 | 1 | 1 | 1 | 1 | 0 | 1 |
| ZINC09387950 | 0 | 0 | 0 | 0 | 1 | 0 | 0 | 0 | 1 | 0 | 1 | 0 | 1 | 3 | 2 | 3 |
| ZINC11937379 | 0 | 1 | 1 | 1 | 0 | 1 | 0 | 0 | 1 | 1 | 1 | 1 | 2 | 0 | 1 | 1 |
| ZINC12128362 | 0 | 0 | 0 | 0 | 0 | 0 | 0 | 0 | 1 | 1 | 1 | 1 | 2 | 2 | 2 | 2 |
| ZINC12318638 | 0 | 0 | 0 | 0 | 1 | 0 | 0 | 0 | 0 | 0 | 0 | 0 | 2 | 3 | 3 | 3 |
| ZINC12974658 | 0 | 0 | 1 | 0 | 0 | 0 | 0 | 0 | 0 | 1 | 0 | 0 | 3 | 2 | 2 | 3 |
| ZINC13133646 | 0 | 0 | 0 | 0 | 0 | 0 | 0 | 0 | 0 | 1 | 1 | 1 | 3 | 2 | 2 | 2 |
| ZINC14459606 | 0 | 1 | 1 | 1 | 0 | 1 | 1 | 1 | 1 | 0 | 1 | 0 | 2 | 1 | 0 | 1 |
| ZINC14991854 | 0 | 0 | 0 | 0 | 1 | 0 | 0 | 0 | 1 | 1 | 1 | 1 | 1 | 2 | 2 | 2 |
| ZINC19836634 | 0 | 0 | 0 | 0 | 1 | 1 | 0 | 0 | 1 | 1 | 1 | 1 | 1 | 1 | 2 | 2 |
| ZINC19973019 | 1 | 0 | 0 | 0 | 1 | 1 | 1 | 1 | 1 | 0 | 1 | 0 | 0 | 2 | 1 | 2 |
| ZINC20465636 | 0 | 0 | 0 | 0 | 0 | 1 | 0 | 0 | 0 | 1 | 0 | 0 | 3 | 1 | 3 | 3 |
| ZINC20727483 | 0 | 0 | 0 | 0 | 0 | 0 | 0 | 0 | 0 | 0 | 0 | 0 | 3 | 3 | 3 | 3 |
| ZINC25423284 | 0 | 0 | 1 | 0 | 0 | 0 | 0 | 0 | 0 | 0 | 0 | 0 | 3 | 3 | 2 | 3 |
| ZINC25558227 | 0 | 0 | 0 | 0 | 1 | 1 | 0 | 0 | 0 | 0 | 1 | 0 | 2 | 2 | 2 | 3 |
| ZINC27938058 | 0 | 0 | 0 | 0 | 0 | 1 | 0 | 0 | 0 | 1 | 0 | 0 | 3 | 1 | 3 | 3 |
| ZINC31794229 | 0 | 0 | 0 | 0 | 0 | 0 | 0 | 0 | 0 | 0 | 0 | 0 | 3 | 3 | 3 | 3 |
| ZINC32479623 | 0 | 0 | 0 | 0 | 0 | 1 | 0 | 0 | 0 | 1 | 0 | 0 | 3 | 1 | 3 | 3 |
| ZINC32676021 | 1 | 0 | 0 | 0 | 0 | 0 | 0 | 0 | 0 | 0 | 0 | 0 | 2 | 3 | 3 | 3 |
| ZINC32889535 | 0 | 0 | 0 | 0 | 1 | 0 | 0 | 0 | 1 | 0 | 0 | 0 | 1 | 3 | 3 | 3 |
| ZINC36083924 | 1 | 0 | 1 | 0 | 1 | 1 | 1 | 1 | 1 | 1 | 1 | 1 | 0 | 1 | 0 | 1 |
| ZINC38828316 | 0 | 1 | 0 | 0 | 0 | 1 | 0 | 0 | 1 | 1 | 0 | 0 | 2 | 0 | 3 | 3 |
| ZINC38933721 | 0 | 1 | 1 | 1 | 1 | 1 | 0 | 0 | 1 | 0 | 1 | 0 | 1 | 1 | 1 | 2 |
| ZINC39936665 | 1 | 0 | 1 | 0 | 0 | 0 | 0 | 0 | 1 | 0 | 1 | 0 | 1 | 3 | 1 | 3 |
| ZINC40754613 | 0 | 0 | 0 | 0 | 1 | 0 | 0 | 0 | 1 | 0 | 1 | 0 | 1 | 3 | 2 | 3 |
| ZINC41007038 | 1 | 0 | 1 | 0 | 0 | 1 | 1 | 1 | 1 | 0 | 1 | 0 | 1 | 2 | 0 | 2 |
| ZINC46671675 | 0 | 0 | 0 | 0 | 0 | 1 | 0 | 0 | 0 | 1 | 0 | 0 | 3 | 1 | 3 | 3 |
| ZINC47321189 | 0 | 0 | 0 | 0 | 0 | 0 | 0 | 0 | 0 | 1 | 0 | 0 | 3 | 2 | 3 | 3 |
| ZINC47652361 | 0 | 0 | 0 | 0 | 0 | 0 | 0 | 0 | 0 | 0 | 0 | 0 | 3 | 3 | 3 | 3 |
| ZINC48909744 | 0 | 0 | 0 | 0 | 1 | 0 | 0 | 0 | 0 | 0 | 0 | 0 | 2 | 3 | 3 | 3 |
| ZINC50732992 | 0 | 0 | 0 | 0 | 0 | 0 | 0 | 0 | 0 | 1 | 0 | 0 | 3 | 2 | 3 | 3 |
| ZINC54847371 | 0 | 0 | 0 | 0 | 0 | 1 | 0 | 0 | 1 | 1 | 0 | 0 | 2 | 1 | 3 | 3 |
| ZINC55780516 | 0 | 0 | 0 | 0 | 0 | 1 | 0 | 0 | 1 | 1 | 1 | 1 | 2 | 1 | 2 | 2 |
| ZINC57500351 | 0 | 0 | 0 | 0 | 0 | 0 | 0 | 0 | 0 | 0 | 0 | 0 | 3 | 3 | 3 | 3 |
| ZINC57832304 | 0 | 0 | 0 | 0 | 0 | 0 | 0 | 0 | 0 | 1 | 0 | 0 | 3 | 2 | 3 | 3 |
| ZINC59422622 | 0 | 0 | 0 | 0 | 1 | 1 | 0 | 0 | 0 | 0 | 1 | 0 | 2 | 2 | 2 | 3 |

**Table S4b.** The comparison of prediction results for randomly collected drugs among four models. “1” refers to active, while “0” refers to inactive. Green refers to the desirable activity, while red refers to the undesirable activity. “R”, “P”, “A” and “P+A” represent REDIAL-2020, the developed predictor COVID-19-CP, Attentive FP model and consensus model obtained from NCATS library, respectively.

| Compounds | 3CL | | | | CPE | | | | AlphaLISA | | | | No. Inactive Predictions | | | |
| --- | --- | --- | --- | --- | --- | --- | --- | --- | --- | --- | --- | --- | --- | --- | --- | --- |
|  | R | P | A | P+A | R | P | A | P+A | R | P | A | P+A | R | P | A | P+A |
| ZINC63280264 | 0 | 0 | 0 | 0 | 0 | 0 | 0 | 0 | 0 | 0 | 0 | 0 | 3 | 3 | 3 | 3 |
| ZINC63385755 | 0 | 0 | 0 | 0 | 0 | 0 | 0 | 0 | 1 | 0 | 0 | 0 | 2 | 3 | 3 | 3 |
| ZINC64568519 | 0 | 1 | 1 | 1 | 0 | 1 | 0 | 0 | 0 | 0 | 0 | 0 | 3 | 1 | 2 | 2 |
| ZINC69744899 | 0 | 0 | 0 | 0 | 1 | 0 | 1 | 0 | 0 | 1 | 0 | 0 | 2 | 2 | 2 | 3 |
| ZINC69753606 | 0 | 0 | 0 | 0 | 0 | 0 | 0 | 0 | 1 | 1 | 0 | 0 | 2 | 2 | 3 | 3 |
| ZINC72144035 | 0 | 0 | 0 | 0 | 0 | 0 | 0 | 0 | 0 | 0 | 0 | 0 | 3 | 3 | 3 | 3 |
| ZINC74577997 | 0 | 0 | 1 | 0 | 0 | 0 | 0 | 0 | 1 | 1 | 0 | 0 | 2 | 2 | 2 | 3 |
| ZINC74794717 | 0 | 0 | 0 | 0 | 0 | 0 | 0 | 0 | 0 | 1 | 0 | 0 | 3 | 2 | 3 | 3 |
| ZINC77181741 | 0 | 0 | 0 | 0 | 0 | 0 | 0 | 0 | 0 | 0 | 0 | 0 | 3 | 3 | 3 | 3 |
| ZINC77512049 | 0 | 0 | 0 | 0 | 1 | 0 | 0 | 0 | 0 | 0 | 0 | 0 | 2 | 3 | 3 | 3 |
| ZINC78466466 | 0 | 0 | 0 | 0 | 0 | 0 | 0 | 0 | 0 | 1 | 0 | 0 | 3 | 2 | 3 | 3 |
| ZINC79205551 | 0 | 0 | 0 | 0 | 1 | 0 | 1 | 0 | 0 | 0 | 0 | 0 | 2 | 3 | 2 | 3 |
| ZINC79752215 | 0 | 0 | 0 | 0 | 0 | 0 | 0 | 0 | 0 | 1 | 0 | 0 | 3 | 2 | 3 | 3 |
| ZINC80072988 | 0 | 0 | 0 | 0 | 0 | 1 | 1 | 1 | 0 | 1 | 0 | 0 | 3 | 1 | 2 | 2 |
| ZINC80191860 | 0 | 0 | 0 | 0 | 0 | 0 | 0 | 0 | 0 | 0 | 0 | 0 | 3 | 3 | 3 | 3 |
| ZINC81048608 | 0 | 0 | 0 | 0 | 0 | 1 | 0 | 0 | 0 | 1 | 0 | 0 | 3 | 1 | 3 | 3 |
| ZINC82125977 | 1 | 0 | 0 | 0 | 0 | 0 | 0 | 0 | 0 | 0 | 0 | 0 | 2 | 3 | 3 | 3 |
| ZINC86011460 | 1 | 1 | 1 | 1 | 0 | 0 | 0 | 0 | 0 | 0 | 0 | 0 | 2 | 2 | 2 | 2 |
| ZINC86210224 | 0 | 0 | 0 | 0 | 0 | 1 | 0 | 0 | 1 | 0 | 0 | 0 | 2 | 2 | 3 | 3 |
| ZINC87105059 | 0 | 0 | 0 | 0 | 1 | 0 | 0 | 0 | 1 | 0 | 0 | 0 | 1 | 3 | 3 | 3 |
| ZINC88777263 | 0 | 0 | 1 | 0 | 1 | 0 | 1 | 0 | 1 | 0 | 1 | 0 | 1 | 3 | 0 | 3 |
| ZINC89642390 | 0 | 0 | 0 | 0 | 1 | 1 | 1 | 1 | 1 | 0 | 1 | 0 | 1 | 2 | 1 | 2 |
| ZINC89667165 | 0 | 0 | 0 | 0 | 1 | 1 | 1 | 1 | 0 | 0 | 0 | 0 | 2 | 2 | 2 | 2 |
| ZINC90688372 | 0 | 1 | 0 | 0 | 0 | 0 | 0 | 0 | 0 | 0 | 0 | 0 | 3 | 2 | 3 | 3 |
| ZINC91437053 | 0 | 0 | 0 | 0 | 0 | 0 | 0 | 0 | 0 | 0 | 0 | 0 | 3 | 3 | 3 | 3 |
| ZINC91461546 | 0 | 0 | 1 | 0 | 1 | 0 | 1 | 0 | 0 | 0 | 0 | 0 | 2 | 3 | 1 | 3 |
| ZINC91491511 | 0 | 0 | 0 | 0 | 1 | 1 | 0 | 0 | 0 | 0 | 0 | 0 | 2 | 2 | 3 | 3 |
| ZINC91723891 | 0 | 0 | 0 | 0 | 0 | 0 | 0 | 0 | 0 | 0 | 0 | 0 | 3 | 3 | 3 | 3 |
| ZINC92003224 | 1 | 0 | 1 | 0 | 0 | 0 | 0 | 0 | 0 | 0 | 0 | 0 | 2 | 3 | 2 | 3 |
| ZINC92175379 | 0 | 0 | 0 | 0 | 0 | 0 | 0 | 0 | 0 | 0 | 0 | 0 | 3 | 3 | 3 | 3 |
| ZINC92378211 | 0 | 0 | 0 | 0 | 0 | 0 | 0 | 0 | 1 | 0 | 0 | 0 | 2 | 3 | 3 | 3 |
| ZINC92423737 | 0 | 0 | 0 | 0 | 1 | 0 | 0 | 0 | 1 | 0 | 0 | 0 | 1 | 3 | 3 | 3 |
| ZINC92511336 | 0 | 0 | 0 | 0 | 0 | 0 | 1 | 0 | 0 | 1 | 0 | 0 | 3 | 2 | 2 | 3 |
| ZINC92567630 | 0 | 0 | 1 | 0 | 0 | 1 | 0 | 0 | 0 | 0 | 1 | 0 | 3 | 2 | 1 | 3 |
| ZINC92585624 | 0 | 0 | 0 | 0 | 0 | 0 | 0 | 0 | 0 | 0 | 0 | 0 | 3 | 3 | 3 | 3 |
| ZINC93105931 | 0 | 0 | 0 | 0 | 0 | 0 | 0 | 0 | 0 | 1 | 0 | 0 | 3 | 2 | 3 | 3 |
| ZINC93255666 | 0 | 0 | 0 | 0 | 0 | 0 | 1 | 0 | 0 | 0 | 1 | 0 | 3 | 3 | 1 | 3 |
| ZINC93292006 | 0 | 1 | 0 | 0 | 1 | 1 | 0 | 0 | 0 | 0 | 0 | 0 | 2 | 1 | 3 | 3 |
| ZINC93584834 | 0 | 0 | 0 | 0 | 0 | 0 | 0 | 0 | 0 | 0 | 0 | 0 | 3 | 3 | 3 | 3 |
| ZINC93765972 | 1 | 0 | 0 | 0 | 0 | 0 | 0 | 0 | 0 | 1 | 0 | 0 | 2 | 2 | 3 | 3 |
| ZINC93825787 | 0 | 0 | 0 | 0 | 1 | 0 | 0 | 0 | 0 | 0 | 0 | 0 | 2 | 3 | 3 | 3 |
| ZINC94004908 | 0 | 0 | 0 | 0 | 0 | 0 | 0 | 0 | 0 | 0 | 0 | 0 | 3 | 3 | 3 | 3 |
| ZINC94205845 | 0 | 0 | 0 | 0 | 0 | 1 | 0 | 0 | 1 | 0 | 1 | 0 | 2 | 2 | 2 | 3 |
| ZINC94231932 | 1 | 0 | 0 | 0 | 0 | 0 | 0 | 0 | 0 | 0 | 0 | 0 | 2 | 3 | 3 | 3 |
| ZINC94410553 | 0 | 0 | 0 | 0 | 0 | 0 | 0 | 0 | 0 | 0 | 0 | 0 | 3 | 3 | 3 | 3 |
| ZINC94558641 | 0 | 0 | 0 | 0 | 0 | 0 | 0 | 0 | 0 | 0 | 0 | 0 | 3 | 3 | 3 | 3 |
| ZINC94613560 | 1 | 0 | 1 | 0 | 1 | 0 | 0 | 0 | 0 | 0 | 0 | 0 | 1 | 3 | 2 | 3 |
| ZINC94782020 | 0 | 0 | 0 | 0 | 1 | 0 | 0 | 0 | 0 | 1 | 0 | 0 | 2 | 2 | 3 | 3 |
| ZINC94851479 | 1 | 0 | 0 | 0 | 0 | 0 | 0 | 0 | 0 | 0 | 0 | 0 | 2 | 3 | 3 | 3 |
| ZINC94943781 | 0 | 0 | 0 | 0 | 0 | 0 | 0 | 0 | 0 | 0 | 0 | 0 | 3 | 3 | 3 | 3 |

**Table S5** The overall performance on the prediction of potential compounds/drugs by REDIAL and COVID-19-CP by incorporating the reconstructed 3CL model. “R”, “P” and “E” represent REDIAL, the developed predictor and experimental activity obtained from NCATS library, respectively.

| Drugs | 3CL | | | CPE | | | cytotox | | | ACE2 | | | AlphaLISA | | | TruHit | | | Accuracy | |
| --- | --- | --- | --- | --- | --- | --- | --- | --- | --- | --- | --- | --- | --- | --- | --- | --- | --- | --- | --- | --- |
|  | R | P | E | R | P | E | R | P | E | R | P | E | R | P | E | R | P | E | R | P |
| Acetaminophen | 1 | 0 | 0 | 0 | 1 | 0 | 1 | 1 | 0 | 0 | 0 | 0 | 0 | 0 | 0 | 0 | 1 | 0 | 50.0% | 67.0% |
| Azithromycin | 0 | 1 | 0 | 1 | 0 | 0 | 0 | 0 | 0 | 0 | 0 | 0 | 0 | 0 | 0 | 0 | 0 | 0 | 33.3% | 66.7% |
| Camostat | 0 | 1 | 0 | 0 | 0 | 0 | 0 | 0 | 0 | 0 | 1 | 0 | 1 | 0 | 0 | 0 | 1 | 0 | 100.0% | 50.0% |
| Chloroquine | 0 | 0 | 0 | 1 | 1 | 1 | 0 | 0 | 0 | 1 | 0 | 0 | 1 | 1 | 0 | 1 | 1 | 0 | 50.0% | 50.0% |
| Colchicine | 0 | 1 | 0 | 0 | 1 | 0 | 1 | 1 | 0 | 1 | 1 | 0 | 0 | 1 | 0 | 0 | 1 | 0 | 50.0% | 50.0% |
| Darunavir | 1 | 0 | 1 | 1 | 0 | 0 | 0 | 1 | 0 | 1 | 1 | 0 | 1 | 0 | 0 | 1 | 0 | 0 | 33.3% | 66.7% |
| Favipiravir | 0 | 0 | 0 | 1 | 0 | 0 | 0 | 0 | 0 | 1 | 0 | 1 | 0 | 0 | 0 | 0 | 1 | 0 | 83.3% | 83.3% |
| Gemcitabine | 0 | 0 | 0 | 0 | 0 | 0 | 0 | 1 | 0 | 0 | 0 | 0 | 0 | 0 | 0 | 0 | 0 | 0 | 100.0% | 83.3% |
| Hydroxychloroquine | 0 | 0 | 0 | 1 | 1 | 0 | 0 | 0 | 0 | 1 | 0 | 0 | 1 | 0 | 0 | 0 | 1 | 0 | 66.7% | 67.0% |
| Indomethacin | 1 | 0 | 1 | 0 | 1 | 0 | 0 | 0 | 0 | 1 | 1 | 1 | 1 | 1 | 1 | 0 | 1 | 0 | 50.0% | 50.0% |
| Ivermectin | 1 | 0 | 0 | 0 | 0 | 1 | 0 | 1 | 0 | 0 | 1 | 0 | 1 | 1 | 1 | 1 | 0 | 0 | 83.3% | 67.0% |
| Loperamide | 0 | 0 | 1 | 1 | 0 | 1 | 1 | 0 | 0 | 0 | 1 | 0 | 1 | 1 | 0 | 0 | 0 | 0 | 33.3% | 50.0% |
| Lopinavir | 0 | 0 | 0 | 1 | 0 | 1 | 1 | 1 | 0 | 0 | 1 | 0 | 1 | 1 | 1 | 1 | 0 | 0 | 83.3% | 50.0% |
| Losartan | 1 | 0 | 0 | 1 | 0 | 0 | 0 | 0 | 0 | 1 | 0 | 0 | 1 | 0 | 1 | 1 | 1 | 0 | 50.0% | 83.3% |
| Mefloquine | 0 | 0 | 0 | 1 | 0 | 1 | 0 | 1 | 0 | 1 | 1 | 0 | 1 | 0 | 0 | 1 | 0 | 0 | 100.0% | 50.0% |
| Nafamostat | 1 | 0 | 0 | 0 | 1 | 0 | 0 | 1 | 0 | 1 | 1 | 0 | 1 | 1 | 0 | 1 | 1 | 0 | 33.3% | 50.0% |
| Nelfinavir | 0 | 1 | 0 | 0 | 0 | 1 | 1 | 0 | 0 | 1 | 1 | 0 | 1 | 1 | 1 | 1 | 1 | 0 | 66.7% | 33.9% |
| Niclosamide | 1 | 1 | 0 | 0 | 0 | 1 | 1 | 0 | 0 | 0 | 0 | 0 | 1 | 0 | 1 | 1 | 0 | 0 | 100% | 83.0% |
| Nitazoxanide | 1 | 0 | 0 | 1 | 1 | 1 | 1 | 1 | 0 | 1 | 1 | 0 | 1 | 0 | 1 | 1 | 1 | 0 | 33.3% | 50.0% |
| Oseltamivir | 0 | 0 | 0 | 0 | 1 | 0 | 0 | 0 | 0 | 0 | 1 | 0 | 0 | 0 | 0 | 0 | 1 | 0 | 33.3% | 50.0% |
| Remdesivir | 0 | 0 | 0 | 1 | 1 | 1 | 0 | 0 | 0 | 1 | 0 | 0 | 1 | 0 | 1 | 1 | 0 | 0 | 66.7% | 83.3% |
| Resveratrol | 1 | 0 | 0 | 0 | 1 | 0 | 0 | 0 | 0 | 1 | 0 | 0 | 0 | 1 | 1 | 1 | 1 | 0 | 50.0% | 83.3% |
| Ribavirin | 0 | 0 | 0 | 0 | 1 | 0 | 0 | 1 | 0 | 0 | 0 | 0 | 0 | 1 | 0 | 0 | 1 | 0 | 33.3% | 67.0% |
| Ritonavir | 0 | 0 | 0 | 0 | 0 | 0 | 1 | 0 | 0 | 1 | 0 | 0 | 1 | 0 | 1 | 1 | 1 | 0 | 50.0% | 66.7% |
| Teicoplanin | 1 | 0 | 1 | 0 | 1 | 1 | 0 | 0 | 0 | 1 | 1 | 0 | 1 | 0 | 1 | 1 | 1 | 0 | 33.3% | 33.3% |
| Thalidomide | 0 | 0 | 0 | 0 | 0 | 0 | 0 | 0 | 0 | 0 | 0 | 0 | 0 | 1 | 0 | 0 | 0 | 0 | 100.0% | 83.3% |
| Tizoxanide | 1 | 0 | 0 | 1 | 1 | 1 | 1 | 1 | 0 | 1 | 0 | 0 | 0 | 1 | 0 | 1 | 1 | 0 | 66.7% | 66.7% |
| Triflupromazine | 0 | 0 | 0 | 1 | 0 | 1 | 1 | 0 | 0 | 0 | 0 | 0 | 1 | 1 | 1 | 1 | 0 | 0 | 66.7% | 83.3% |

**Table S6** The prediction results by the reconstructed 3CL model for active compounds in 3CL assay. “R” and “P” represent REDIAL and the developed predictor, respectively.

| Drug | R | P |
| --- | --- | --- |
| Baicalein | 1 | 1 |
| Benserazide Hydrochloride | 1 | 1 |
| Bonaphthone | 1 | 0 |
| Bronopol | 1 | 1 |
| Carmofur | 0 | 0 |
| Dihydrexidine | 1 | 0 |
| Disulfiram | 1 | 0 |
| Felbinac Ethyl | 0 | 1 |
| MG-132 | 1 | 0 |
| ML311 | 0 | 0 |
| MLS0315771 | 0 | 0 |
| Myricetin | 1 | 0 |
| Oltipraz | 0 | 1 |
| PR-619 | 1 | 1 |
| Proflavine Hemisulfate | 1 | 0 |
| SKF-38393 | 1 | 1 |
| TDZD-8 | 0 | 0 |
| Thioguanosine | 0 | 0 |
| Tideglusib | 1 | 0 |
| WAY-308264 | 0 | 0 |
| Z-DEVD-FMK | 1 | 1 |

**Table S7** The overall performance on the prediction of screening compounsd by REDIAL and COVID-19-CP by incorporating the reconstructed 3CL model. “R” and “P” represent REDIAL and the developed predictor, respectively.

| Compounds | 3CL | | CPE | | AlphaLISA | | No. Inactive Predictions | |
| --- | --- | --- | --- | --- | --- | --- | --- | --- |
|  | R | P | R | P | R | P | R | P |
| ZINC00927555 | 0 | 0 | 1 | 0 | 1 | 0 | 1 | 3 |
| ZINC01669393 | 1 | 0 | 1 | 1 | 1 | 1 | 0 | 1 |
| ZINC01835455 | 0 | 1 | 0 | 1 | 1 | 1 | 2 | 0 |
| ZINC02038278 | 1 | 0 | 0 | 0 | 0 | 0 | 2 | 3 |
| ZINC02245307 | 1 | 0 | 0 | 0 | 0 | 0 | 2 | 3 |
| ZINC03049271 | 1 | 0 | 0 | 0 | 1 | 0 | 1 | 3 |
| ZINC04503565 | 0 | 0 | 0 | 0 | 0 | 0 | 3 | 3 |
| ZINC05626166 | 0 | 0 | 0 | 0 | 0 | 1 | 3 | 2 |
| ZINC06198146 | 0 | 0 | 0 | 0 | 1 | 0 | 2 | 3 |
| ZINC06637259 | 0 | 0 | 0 | 0 | 0 | 1 | 3 | 2 |
| ZINC06805870 | 0 | 0 | 1 | 1 | 0 | 1 | 2 | 1 |
| ZINC08063873 | 0 | 1 | 1 | 1 | 1 | 0 | 1 | 1 |
| ZINC08849480 | 0 | 0 | 1 | 1 | 0 | 1 | 2 | 1 |
| ZINC08857316 | 1 | 1 | 1 | 1 | 1 | 1 | 0 | 0 |
| ZINC09241936 | 0 | 1 | 1 | 1 | 1 | 1 | 1 | 0 |
| ZINC09387950 | 0 | 0 | 1 | 0 | 1 | 0 | 1 | 3 |
| ZINC11937379 | 0 | 0 | 0 | 1 | 1 | 1 | 2 | 1 |
| ZINC12128362 | 0 | 0 | 0 | 0 | 1 | 1 | 2 | 2 |
| ZINC12318638 | 0 | 0 | 1 | 0 | 0 | 0 | 2 | 3 |
| ZINC12974658 | 0 | 0 | 0 | 0 | 0 | 1 | 3 | 2 |
| ZINC13133646 | 0 | 0 | 0 | 0 | 0 | 1 | 3 | 2 |
| ZINC14459606 | 0 | 0 | 0 | 1 | 1 | 0 | 2 | 2 |
| ZINC14991854 | 0 | 0 | 1 | 0 | 1 | 1 | 1 | 2 |
| ZINC19836634 | 0 | 0 | 1 | 1 | 1 | 1 | 1 | 1 |
| ZINC19973019 | 1 | 0 | 1 | 1 | 1 | 0 | 0 | 2 |
| ZINC20465636 | 0 | 0 | 0 | 1 | 0 | 1 | 3 | 1 |
| ZINC20727483 | 0 | 0 | 0 | 0 | 0 | 0 | 3 | 3 |
| ZINC25423284 | 0 | 0 | 0 | 0 | 0 | 0 | 3 | 3 |
| ZINC25558227 | 0 | 1 | 1 | 1 | 0 | 0 | 2 | 1 |
| ZINC27938058 | 0 | 0 | 0 | 1 | 0 | 1 | 3 | 1 |
| ZINC31794229 | 0 | 0 | 0 | 0 | 0 | 0 | 3 | 3 |
| ZINC32479623 | 0 | 0 | 0 | 1 | 0 | 1 | 3 | 1 |
| ZINC32676021 | 1 | 0 | 0 | 0 | 0 | 0 | 2 | 3 |
| ZINC32889535 | 0 | 0 | 1 | 0 | 1 | 0 | 1 | 3 |
| ZINC36083924 | 1 | 1 | 1 | 1 | 1 | 1 | 0 | 0 |
| ZINC38828316 | 0 | 0 | 0 | 1 | 1 | 1 | 2 | 1 |
| ZINC38933721 | 0 | 1 | 1 | 1 | 1 | 0 | 1 | 1 |
| ZINC39936665 | 1 | 0 | 0 | 0 | 1 | 0 | 1 | 3 |
| ZINC40754613 | 0 | 0 | 1 | 0 | 1 | 0 | 1 | 3 |
| ZINC41007038 | 1 | 0 | 0 | 1 | 1 | 0 | 1 | 2 |
| ZINC46671675 | 0 | 0 | 0 | 1 | 0 | 1 | 3 | 1 |
| ZINC47321189 | 0 | 1 | 0 | 0 | 0 | 1 | 3 | 1 |
| ZINC47652361 | 0 | 1 | 0 | 0 | 0 | 0 | 3 | 2 |
| ZINC48909744 | 0 | 0 | 1 | 0 | 0 | 0 | 2 | 3 |
| ZINC50732992 | 0 | 0 | 0 | 0 | 0 | 1 | 3 | 2 |
| ZINC54847371 | 0 | 1 | 0 | 1 | 1 | 1 | 2 | 0 |
| ZINC55780516 | 0 | 0 | 0 | 1 | 1 | 1 | 2 | 1 |
| ZINC57500351 | 0 | 1 | 0 | 0 | 0 | 0 | 3 | 2 |
| ZINC57832304 | 0 | 0 | 0 | 0 | 0 | 1 | 3 | 2 |
| ZINC59422622 | 0 | 0 | 1 | 1 | 0 | 0 | 2 | 2 |

**Table S8**. Description of input GAFF features. Feature ID is the ID shown in GAFF+RDKit features, while GAFF ID represents the real order in GAFF features. EW: electron-withdraw group.

| Feature ID | GAFF ID | Atom type | Description | Radius | Coefficient |
| --- | --- | --- | --- | --- | --- |
| 0 | 1 | h1 | H on aliphatic C with 1 EW group | 1.2 | 0.155519 |
| 1 | 2 | h2 | H on aliphatic C with 2 EW group | 1.2 | 0.157683 |
| 2 | 3 | h3 | H on aliphatic C with 3 EW group | 1.2 | 0.11561 |
| 3 | 4 | h4 | H on aliphatic C with 4 EW group | 1.2 | 0.243571 |
| 4 | 5 | h5 | H on aliphatic C with 5 EW group | 1.2 | 0.242485 |
| 5 | 6 | ha | H on aromatic C | 1.2 | 0.20587 |
| 6 | 7 | hc | H on aliphatic C | 1.2 | 0.161566 |
| 7 | 8 | hn | H on N | 1.2 | 0.261173 |
| 8 | 9 | ho | H on O | 1.2 | 0.304896 |
| 9 | 10 | hs | H on S | 1.2 | 0.050542 |
| 10 | 11 | hp | H on P | 1.2 | 0.074645 |
| 11 | 12 | c | sp2 C in C=O, C=S | 1.74 | 0.34419 |
| 12 | 13 | c1 | sp1 C | 1.74 | 0.260395 |
| 13 | 14 | c2 | sp2 C, aliphatic | 1.74 | 0.271417 |
| 14 | 15 | c3 | sp3 C | 1.74 | 0.485916 |
| 15 | 16 | ca | sp2 C, aromatic | 1.74 | 0.219094 |
| 16 | 17 | cc | inner sp2 C in conjugated ring systems | 1.74 | 0.22704 |
| 17 | 18 | ce | inner sp2 C in conjugated chain systems | 1.74 | 0.23027 |
| 18 | 19 | cg | Inner sp1 C in conjugated ring systems | 1.74 | 0.258611 |
| 19 | 20 | cp | bridge aromatic C | 1.74 | 0.038753 |
| 20 | 21 | n | aromatic nitrogen | 1.54 | 0.045682 |
| 21 | 22 | n1 | sp1 N | 1.54 | 0.336189 |
| 22 | 23 | n2 | sp2 N with 2 substituted double bond | 1.54 | 0.349493 |
| 23 | 24 | n3 | sp3 N with 3 substituted | 1.54 | 0.279662 |
| 24 | 25 | n4 | sp3 N with 4 substituted | 1.54 | 0.124458 |
| 25 | 26 | na | sp2 N with 3 substituted | 1.54 | 0.262997 |
| 26 | 27 | nb | inner sp2 N in conjugated ring systems | 1.54 | 0.321079 |
| 27 | 28 | nc | inner sp2 N in conjugated chain systems | 1.54 | 0.375652 |
| 28 | 29 | ne |  | 1.54 | 0.337318 |
| 29 | 30 | nh | amine N connected to the aromatic rings | 1.54 | 0.243705 |
| 30 | 31 | no | N in nitro group | 1.54 | 0.490527 |
| 31 | 32 | o | sp2 O in C=O, COO- | 1.4 | 0.36226 |
| 32 | 33 | oh | sp3 O in hydroxyl group | 1.4 | 0.331556 |
| 33 | 34 | os | sp3 O in ether and ester | 1.4 | 0.323638 |
| 34 | 35 | s |  | 2 | 0.268957 |
| 35 | 36 | s4 | hypervalent S, 3 substituted | 2 | 0.428026 |
| 36 | 37 | s6 | hypervalent S, 4 substituted | 2 | 0.428617 |
| 37 | 38 | sh | sp3 S in thiol group | 2 | 0.370181 |
| 38 | 39 | ss | sp3 S in -SR and SS | 2 | 0.367816 |
| 39 | 40 | p2 | sp2 P (C=P etc) | 2 | 0.362807 |
| 40 | 41 | p3 | sp3 P, 3 subst. | 2 | 0.428887 |
| 41 | 42 | p5 | hypervalent P, 4 substituted | 2 | 0.407535 |
| 42 | 43 | f | any fluorine | 1.6 | 0.359927 |
| 43 | 44 | cl | any chlorine | 1.79 | 0.334519 |
| 44 | 45 | br | any bromine | 2.04 | 0.327391 |
| 45 | 46 | i | any iodine | 2.15 | 0.318243 |
| 46 | 47 | Total number of atoms | | | |

**Table S9**. Description of input RDKit features. Feature ID is the ID shown in GAFF+RDKit features, while RDKit ID represents the real order in RDKit features.

| Feature ID | RDKit ID | Parameter |
| --- | --- | --- |
| 47 | 1 | MaxEStateIndex |
| 48 | 2 | MinEStateIndex |
| 49 | 3 | MaxAbsEStateIndex |
| 50 | 4 | MinAbsEStateIndex |
| 51 | 5 | qed |
| 52 | 6 | MolWt |
| 53 | 7 | HeavyAtomMolWt |
| 54 | 8 | ExactMolWt |
| 55 | 9 | NumValenceElectrons |
| 56 | 10 | NumRadicalElectrons |
| 57 | 11 | MaxPartialCharge |
| 58 | 12 | MinPartialCharge |
| 59 | 13 | MaxAbsPartialCharge |
| 60 | 14 | MinAbsPartialCharge |
| 61 | 15 | FpDensityMorgan1 |
| 62 | 16 | FpDensityMorgan2 |
| 63 | 17 | FpDensityMorgan3 |
| 64 | 18 | BCUT2D_MWHI |
| 65 | 19 | BCUT2D_MWLOW |
| 66 | 20 | BCUT2D_CHGHI |
| 67 | 21 | BCUT2D_CHGLO |
| 68 | 22 | BCUT2D_LOGPHI |
| 69 | 23 | BCUT2D_LOGPLOW |
| 70 | 24 | BCUT2D_MRHI |
| 71 | 25 | BCUT2D_MRLOW |
| 72 | 26 | BalabanJ |
| 73 | 27 | BertzCT |
| 74 | 28 | Chi0 |
| 75 | 29 | Chi0n |
| 76 | 30 | Chi0v |
| 77 | 31 | Chi1 |
| 78 | 32 | Chi1n |
| 79 | 33 | Chi1v |
| 80 | 34 | Chi2n |
| 81 | 35 | Chi2v |
| 82 | 36 | Chi3n |
| 83 | 37 | Chi3v |
| 84 | 38 | Chi4n |
| 85 | 39 | Chi4v |
| 86 | 40 | HallKierAlpha |
| 87 | 41 | Ipc |
| 88 | 42 | Kappa1 |
| 89 | 43 | Kappa2 |
| 90 | 44 | Kappa3 |
| 91 | 45 | LabuteASA |
| 92 | 46 | PEOE_VSA1 |
| 93 | 47 | PEOE_VSA10 |
| 94 | 48 | PEOE_VSA11 |
| 95 | 49 | PEOE_VSA12 |
| 96 | 50 | PEOE_VSA13 |
| 97 | 51 | PEOE_VSA14 |
| 98 | 52 | PEOE_VSA2 |
| 99 | 53 | PEOE_VSA3 |
| 100 | 54 | PEOE_VSA4 |
| 101 | 55 | PEOE_VSA5 |
| 102 | 56 | PEOE_VSA6 |
| 103 | 57 | PEOE_VSA7 |
| 104 | 58 | PEOE_VSA8 |
| 105 | 59 | PEOE_VSA9 |
| 106 | 60 | SMR_VSA1 |
| 107 | 61 | SMR_VSA10 |
| 108 | 62 | SMR_VSA2 |
| 109 | 63 | SMR_VSA3 |
| 110 | 64 | SMR_VSA4 |
| 111 | 65 | SMR_VSA5 |
| 112 | 66 | SMR_VSA6 |
| 113 | 67 | SMR_VSA7 |
| 114 | 68 | SMR_VSA8 |
| 115 | 69 | SMR_VSA9 |
| 116 | 70 | SlogP_VSA1 |
| 117 | 71 | SlogP_VSA10 |
| 118 | 72 | SlogP_VSA11 |
| 119 | 73 | SlogP_VSA12 |
| 120 | 74 | SlogP_VSA2 |
| 121 | 75 | SlogP_VSA3 |
| 122 | 76 | SlogP_VSA4 |
| 123 | 77 | SlogP_VSA5 |
| 124 | 78 | SlogP_VSA6 |
| 125 | 79 | SlogP_VSA7 |
| 126 | 80 | SlogP_VSA8 |
| 127 | 81 | SlogP_VSA9 |
| 128 | 82 | TPSA |
| 129 | 83 | EState_VSA1 |
| 130 | 84 | EState_VSA10 |
| 131 | 85 | EState_VSA11 |
| 132 | 86 | EState_VSA2 |
| 133 | 87 | EState_VSA3 |
| 134 | 88 | EState_VSA4 |
| 135 | 89 | EState_VSA5 |
| 136 | 90 | EState_VSA6 |
| 137 | 91 | EState_VSA7 |
| 138 | 92 | EState_VSA8 |
| 139 | 93 | EState_VSA9 |
| 140 | 94 | VSA_EState1 |
| 141 | 95 | VSA_EState10 |
| 142 | 96 | VSA_EState2 |
| 143 | 97 | VSA_EState3 |
| 144 | 98 | VSA_EState4 |
| 145 | 99 | VSA_EState5 |
| 146 | 100 | VSA_EState6 |
| 147 | 101 | VSA_EState7 |
| 148 | 102 | VSA_EState8 |
| 149 | 103 | VSA_EState9 |
| 150 | 104 | FractionCSP3 |
| 151 | 105 | HeavyAtomCount |
| 152 | 106 | NHOHCount |
| 153 | 107 | NOCount |
| 154 | 108 | NumAliphaticCarbocycles |
| 155 | 109 | NumAliphaticHeterocycles |
| 156 | 110 | NumAliphaticRings |
| 157 | 111 | NumAromaticCarbocycles |
| 158 | 112 | NumAromaticHeterocycles |
| 159 | 113 | NumAromaticRings |
| 160 | 114 | NumHAcceptors |
| 161 | 115 | NumHDonors |
| 162 | 116 | NumHeteroatoms |
| 163 | 117 | NumRotatableBonds |
| 164 | 118 | NumSaturatedCarbocycles |
| 165 | 119 | NumSaturatedHeterocycles |
| 166 | 120 | NumSaturatedRings |
| 167 | 121 | RingCount |
| 168 | 122 | MolLogP |
| 169 | 123 | MolMR |
| 170 | 124 | fr_Al_COO |
| 171 | 125 | fr_Al_OH |
| 172 | 126 | fr_Al_OH_noTert |
| 173 | 127 | fr_ArN |
| 174 | 128 | fr_Ar_COO |
| 175 | 129 | fr_Ar_N |
| 176 | 130 | fr_Ar_NH |
| 177 | 131 | fr_Ar_OH |
| 178 | 132 | fr_COO |
| 179 | 133 | fr_COO2 |
| 180 | 134 | fr_C_O |
| 181 | 135 | fr_C_O_noCOO |
| 182 | 136 | fr_C_S |
| 183 | 137 | fr_HOCCN |
| 184 | 138 | fr_Imine |
| 185 | 139 | fr_NH0 |
| 186 | 140 | fr_NH1 |
| 187 | 141 | fr_NH2 |
| 188 | 142 | fr_N_O |
| 189 | 143 | fr_Ndealkylation1 |
| 190 | 144 | fr_Ndealkylation2 |
| 191 | 145 | fr_Nhpyrrole |
| 192 | 146 | fr_SH |
| 193 | 147 | fr_aldehyde |
| 194 | 148 | fr_alkyl_carbamate |
| 195 | 149 | fr_alkyl_halide |
| 196 | 150 | fr_allylic_oxid |
| 197 | 151 | fr_amide |
| 198 | 152 | fr_amidine |
| 199 | 153 | fr_aniline |
| 200 | 154 | fr_aryl_methyl |
| 201 | 155 | fr_azide |
| 202 | 156 | fr_azo |
| 203 | 157 | fr_barbitur |
| 204 | 158 | fr_benzene |
| 205 | 159 | fr_benzodiazepine |
| 206 | 160 | fr_bicyclic |
| 207 | 161 | fr_diazo |
| 208 | 162 | fr_dihydropyridine |
| 209 | 163 | fr_epoxide |
| 210 | 164 | fr_ester |
| 211 | 165 | fr_ether |
| 212 | 166 | fr_furan |
| 213 | 167 | fr_guanido |
| 214 | 168 | fr_halogen |
| 215 | 169 | fr_hdrzine |
| 216 | 170 | fr_hdrzone |
| 217 | 171 | fr_imidazole |
| 218 | 172 | fr_imide |
| 219 | 173 | fr_isocyan |
| 220 | 174 | fr_isothiocyan |
| 221 | 175 | fr_ketone |
| 222 | 176 | fr_ketone_Topliss |
| 223 | 177 | fr_lactam |
| 224 | 178 | fr_lactone |
| 225 | 179 | fr_methoxy |
| 226 | 180 | fr_morpholine |
| 227 | 181 | fr_nitrile |
| 228 | 182 | fr_nitro |
| 229 | 183 | fr_nitro_arom |
| 230 | 184 | fr_nitro_arom_nonortho |
| 231 | 185 | fr_nitroso |
| 232 | 186 | fr_oxazole |
| 233 | 187 | fr_oxime |
| 234 | 188 | fr_para_hydroxylation |
| 235 | 189 | fr_phenol |
| 236 | 190 | fr_phenol_noOrthoHbond |
| 237 | 191 | fr_phos_acid |
| 238 | 192 | fr_phos_ester |
| 239 | 193 | fr_piperdine |
| 240 | 194 | fr_piperzine |
| 241 | 195 | fr_priamide |
| 242 | 196 | fr_prisulfonamd |
| 243 | 197 | fr_pyridine |
| 244 | 198 | fr_quatN |
| 245 | 199 | fr_sulfide |
| 246 | 200 | fr_sulfonamd |
| 247 | 201 | fr_sulfone |
| 248 | 202 | fr_term_acetylene |
| 249 | 203 | fr_tetrazole |
| 250 | 204 | fr_thiazole |
| 251 | 205 | fr_thiocyan |
| 252 | 206 | fr_thiophene |
| 253 | 207 | fr_unbrch_alkane |
| 254 | 208 | fr_urea |

**Table S10** Scores of metrics of each subset for Attentive FP models in 9 assays. 0-9 represent the divided subsets for each group of actives and inactives.

1. 3CL assay

| Datasets | Metrics | 0 | 1 | 2 | 3 | 4 |
| --- | --- | --- | --- | --- | --- | --- |
| Validation | AUC | 0.78 | 0.79 | 0.76 | 0.74 | 0.77 |
|  | ACC | 0.69 | 0.7 | 0.65 | 0.64 | 0.7 |
|  | F1 | 0.67 | 0.59 | 0.62 | 0.6 | 0.63 |
|  | PRE | 0.57 | 0.64 | 0.54 | 0.54 | 0.63 |
|  | REC | 0.82 | 0.55 | 0.74 | 0.67 | 0.64 |
| Test | AUC | 0.66 | 0.66 | 0.62 | 0.64 | 0.66 |
|  | ACC | 0.59 | 0.63 | 0.63 | 0.63 | 0.62 |
|  | F1 | 0.63 | 0.57 | 0.66 | 0.65 | 0.59 |
|  | PRE | 0.58 | 0.67 | 0.62 | 0.63 | 0.64 |
|  | REC | 0.68 | 0.5 | 0.7 | 0.67 | 0.55 |

1. CPE assay

| Datasets | Metrics | 0 | 1 | 2 | 3 | 4 |
| --- | --- | --- | --- | --- | --- | --- |
| Validation | AUC | 0.77 | 0.74 | 0.75 | 0.77 | 0.84 |
|  | ACC | 0.68 | 0.68 | 0.7 | 0.7 | 0.74 |
|  | F1 | 0.69 | 0.66 | 0.62 | 0.68 | 0.71 |
|  | PRE | 0.64 | 0.65 | 0.75 | 0.68 | 0.74 |
|  | REC | 0.76 | 0.67 | 0.54 | 0.67 | 0.69 |
| Test | AUC | 0.8 | 0.78 | 0.76 | 0.78 | 0.8 |
|  | ACC | 0.74 | 0.74 | 0.7 | 0.71 | 0.73 |
|  | F1 | 0.76 | 0.75 | 0.66 | 0.69 | 0.71 |
|  | PRE | 0.71 | 0.74 | 0.76 | 0.73 | 0.75 |
|  | REC | 0.82 | 0.76 | 0.57 | 0.66 | 0.67 |

1. ACE2 assay

| Datasets | Metrics | 0 | 1 | 2 | 3 | 4 | 5 | 6 | 7 | 8 | 9 |
| --- | --- | --- | --- | --- | --- | --- | --- | --- | --- | --- | --- |
| Validation | AUC | 0.86 | 0.85 | 0.61 | 0.83 | 0.76 | 0.74 | 0.74 | 0.77 | 0.77 | 0.84 |
|  | ACC | 0.71 | 0.76 | 0.65 | 0.71 | 0.65 | 0.59 | 0.59 | 0.56 | 0.71 | 0.71 |
|  | F1 | 0.72 | 0.85 | 0.78 | 0.76 | 0.7 | 0.61 | 0.61 | 0.52 | 0.76 | 0.78 |
|  | PRE | 1 | 0.76 | 0.68 | 0.84 | 0.82 | 0.85 | 0.85 | 1 | 0.84 | 0.78 |
|  | REC | 0.57 | 0.96 | 0.91 | 0.7 | 0.61 | 0.48 | 0.48 | 0.35 | 0.7 | 0.78 |
| Test | AUC | 0.67 | 0.61 | 0.68 | 0.73 | 0.76 | 0.69 | 0.58 | 0.57 | 0.61 | 0.66 |
|  | ACC | 0.68 | 0.54 | 0.58 | 0.65 | 0.64 | 0.67 | 0.63 | 0.63 | 0.64 | 0.53 |
|  | F1 | 0.6 | 0.67 | 0.7 | 0.63 | 0.58 | 0.58 | 0.49 | 0.49 | 0.61 | 0.55 |
|  | PRE | 0.79 | 0.52 | 0.54 | 0.68 | 0.7 | 0.78 | 0.78 | 0.78 | 0.67 | 0.52 |
|  | REC | 0.49 | 0.92 | 0.97 | 0.59 | 0.49 | 0.46 | 0.36 | 0.36 | 0.56 | 0.59 |

1. AlphaLISA assay

| Datasets | Metrics | 0 | 1 |
| --- | --- | --- | --- |
| Validation | AUC | 0.81 | 0.8 |
|  | ACC | 0.73 | 0.7 |
|  | F1 | 0.73 | 0.7 |
|  | PRE | 0.69 | 0.66 |
|  | REC | 0.77 | 0.74 |
| Test | AUC | 0.87 | 0.84 |
|  | ACC | 0.78 | 0.76 |
|  | F1 | 0.79 | 0.77 |
|  | PRE | 0.77 | 0.74 |
|  | REC | 0.82 | 0.82 |

1. TMPRSS2 assay

| Datasets | Metrics | 0 | 1 | 2 | 3 | 4 | 5 | 6 | 7 | 8 | 9 |
| --- | --- | --- | --- | --- | --- | --- | --- | --- | --- | --- | --- |
| Validation | AUC | 0.78 | 0.76 | 0.83 | 0.68 | 0.77 | 0.77 | 0.73 | 0.65 | 0.65 | 0.81 |
|  | ACC | 0.68 | 0.71 | 0.68 | 0.56 | 0.71 | 0.65 | 0.74 | 0.65 | 0.62 | 0.76 |
|  | F1 | 0.69 | 0.75 | 0.72 | 0.65 | 0.75 | 0.7 | 0.78 | 0.67 | 0.61 | 0.75 |
|  | PRE | 0.67 | 0.65 | 0.64 | 0.54 | 0.65 | 0.61 | 0.67 | 0.63 | 0.63 | 0.8 |
|  | REC | 0.71 | 0.88 | 0.82 | 0.82 | 0.88 | 0.82 | 0.94 | 0.71 | 0.59 | 0.71 |
| Test | AUC | 0.72 | 0.68 | 0.77 | 0.59 | 0.7 | 0.73 | 0.69 | 0.63 | 0.73 | 0.74 |
|  | ACC | 0.66 | 0.63 | 0.62 | 0.55 | 0.61 | 0.62 | 0.66 | 0.63 | 0.64 | 0.68 |
|  | F1 | 0.7 | 0.66 | 0.68 | 0.65 | 0.65 | 0.67 | 0.72 | 0.62 | 0.65 | 0.66 |
|  | PRE | 0.62 | 0.61 | 0.58 | 0.53 | 0.58 | 0.59 | 0.61 | 0.64 | 0.64 | 0.72 |
|  | REC | 0.82 | 0.71 | 0.82 | 0.82 | 0.74 | 0.76 | 0.87 | 0.61 | 0.66 | 0.61 |

1. cytotox assay

| Datasets | Metrics | 0 | 1 | 2 | 3 | 4 |
| --- | --- | --- | --- | --- | --- | --- |
| Validation | AUC | 0.84 | 0.82 | 0.84 | 0.81 | 0.82 |
|  | ACC | 0.74 | 0.73 | 0.77 | 0.74 | 0.72 |
|  | F1 | 0.7 | 0.69 | 0.75 | 0.71 | 0.7 |
|  | PRE | 0.8 | 0.79 | 0.79 | 0.76 | 0.7 |
|  | REC | 0.63 | 0.62 | 0.71 | 0.67 | 0.7 |
| Test | AUC | 0.81 | 0.79 | 0.83 | 0.83 | 0.84 |
|  | ACC | 0.72 | 0.71 | 0.73 | 0.74 | 0.76 |
|  | F1 | 0.69 | 0.68 | 0.72 | 0.72 | 0.75 |
|  | PRE | 0.78 | 0.76 | 0.76 | 0.81 | 0.78 |
|  | REC | 0.62 | 0.61 | 0.68 | 0.64 | 0.73 |

1. TruHit assay

| Datasets | Metrics | 0 | 1 |
| --- | --- | --- | --- |
| Validation | AUC | 0.9 | 0.9 |
|  | ACC | 0.8 | 0.79 |
|  | F1 | 0.78 | 0.72 |
|  | PRE | 0.7 | 0.82 |
|  | REC | 0.87 | 0.64 |
| Test | AUC | 0.87 | 0.88 |
|  | ACC | 0.78 | 0.76 |
|  | F1 | 0.8 | 0.71 |
|  | PRE | 0.75 | 0.89 |
|  | REC | 0.84 | 0.6 |

1. HEK293 assay

| Datasets | Metrics | 0 |
| --- | --- | --- |
| Validation | AUC | 0.84 |
|  | ACC | 0.76 |
|  | F1 | 0.71 |
|  | PRE | 0.73 |
|  | REC | 0.69 |
| Test | AUC | 0.82 |
|  | ACC | 0.75 |
|  | F1 | 0.73 |
|  | PRE | 0.78 |
|  | REC | 0.68 |

1. Fibroblast assay

| Datasets | Metrics | 0 | 1 | 2 | 3 | 4 | 5 | 6 | 7 | 8 | 9 |
| --- | --- | --- | --- | --- | --- | --- | --- | --- | --- | --- | --- |
| Validation | AUC | 0.85 | 0.84 | 0.86 | 0.78 | 0.86 | 0.8 | 0.83 | 0.77 | 0.82 | 0.8 |
|  | ACC | 0.76 | 0.74 | 0.8 | 0.74 | 0.8 | 0.76 | 0.74 | 0.68 | 0.76 | 0.73 |
|  | F1 | 0.83 | 0.81 | 0.84 | 0.78 | 0.83 | 0.78 | 0.79 | 0.68 | 0.8 | 0.76 |
|  | PRE | 0.76 | 0.75 | 0.88 | 0.75 | 0.83 | 0.82 | 0.74 | 0.82 | 0.77 | 0.77 |
|  | REC | 0.91 | 0.88 | 0.79 | 0.81 | 0.83 | 0.75 | 0.85 | 0.58 | 0.83 | 0.75 |
| Test | AUC | 0.73 | 0.74 | 0.74 | 0.73 | 0.75 | 0.73 | 0.75 | 0.75 | 0.71 | 0.72 |
|  | ACC | 0.68 | 0.69 | 0.68 | 0.71 | 0.71 | 0.7 | 0.7 | 0.64 | 0.66 | 0.68 |
|  | F1 | 0.73 | 0.73 | 0.69 | 0.73 | 0.71 | 0.71 | 0.73 | 0.55 | 0.66 | 0.68 |
|  | PRE | 0.63 | 0.65 | 0.68 | 0.68 | 0.7 | 0.68 | 0.66 | 0.72 | 0.67 | 0.67 |
|  | REC | 0.86 | 0.82 | 0.7 | 0.78 | 0.71 | 0.75 | 0.82 | 0.44 | 0.65 | 0.69 |


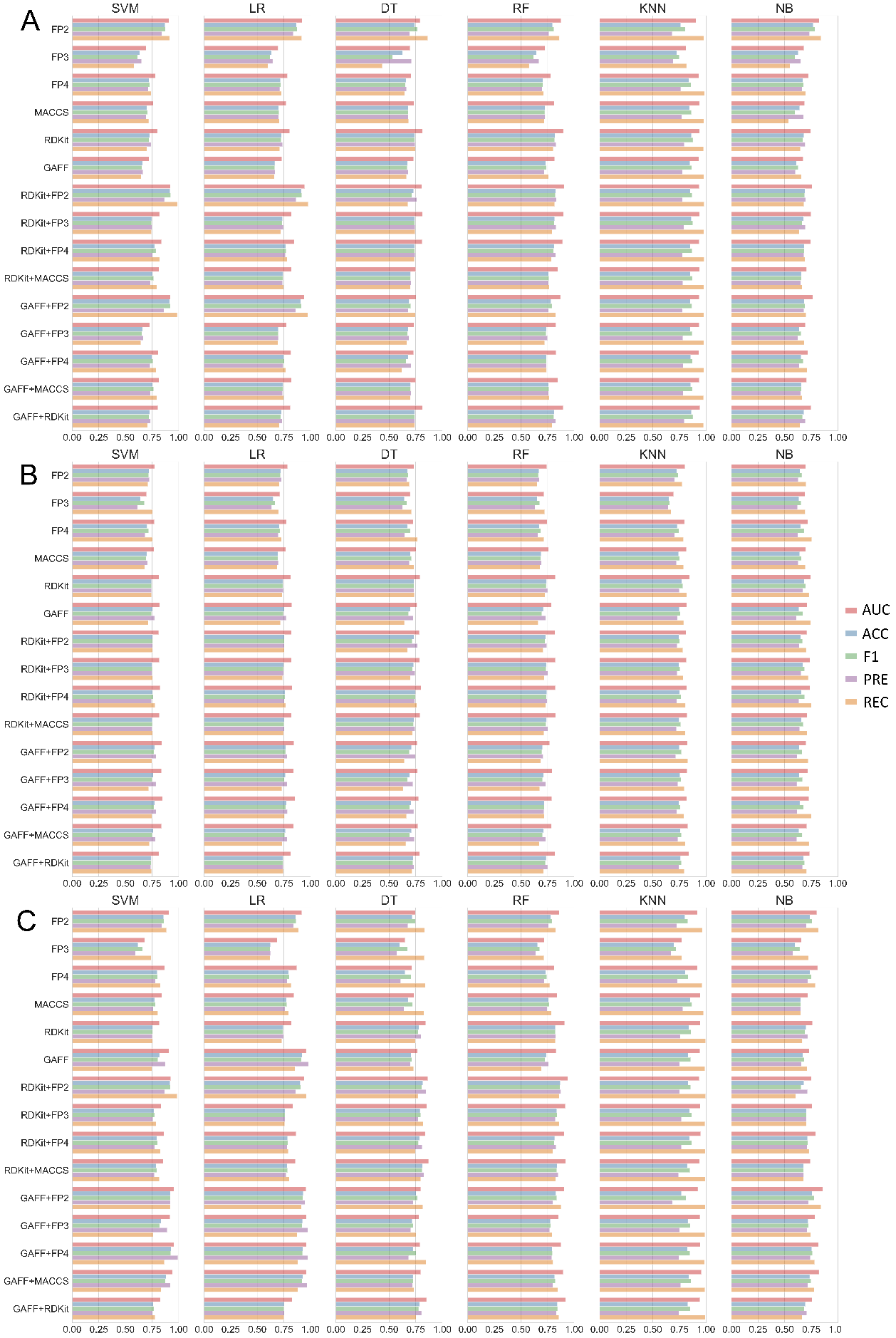


**Figure S1A** Model performance of validation sets for screening assays. A. 3CL, B. HEK293, C. Fibroblast.


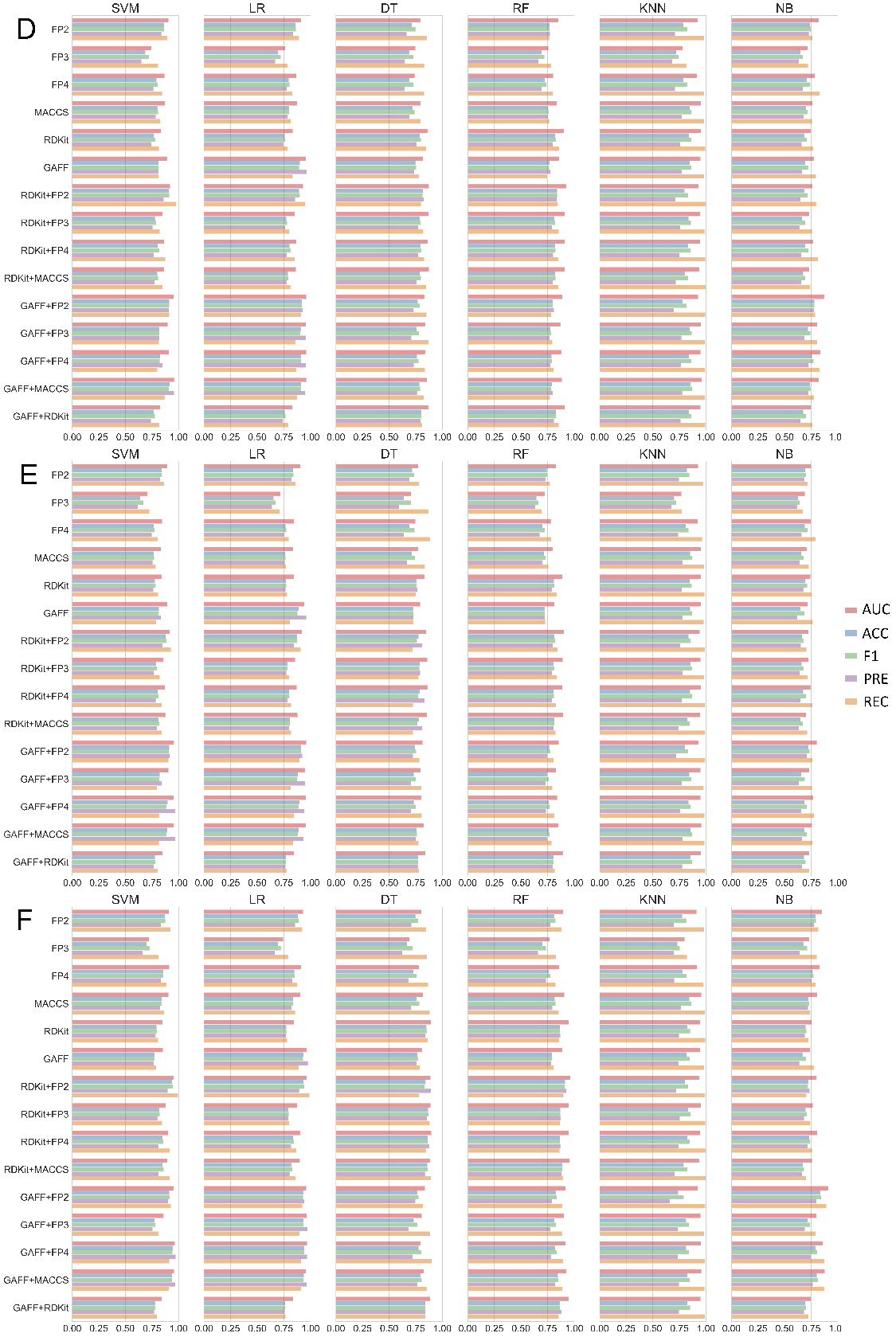


**Figure S1B** Model performance of validation sets for screening assays. D. CPE, E. cytotox, F. ACE2.

**
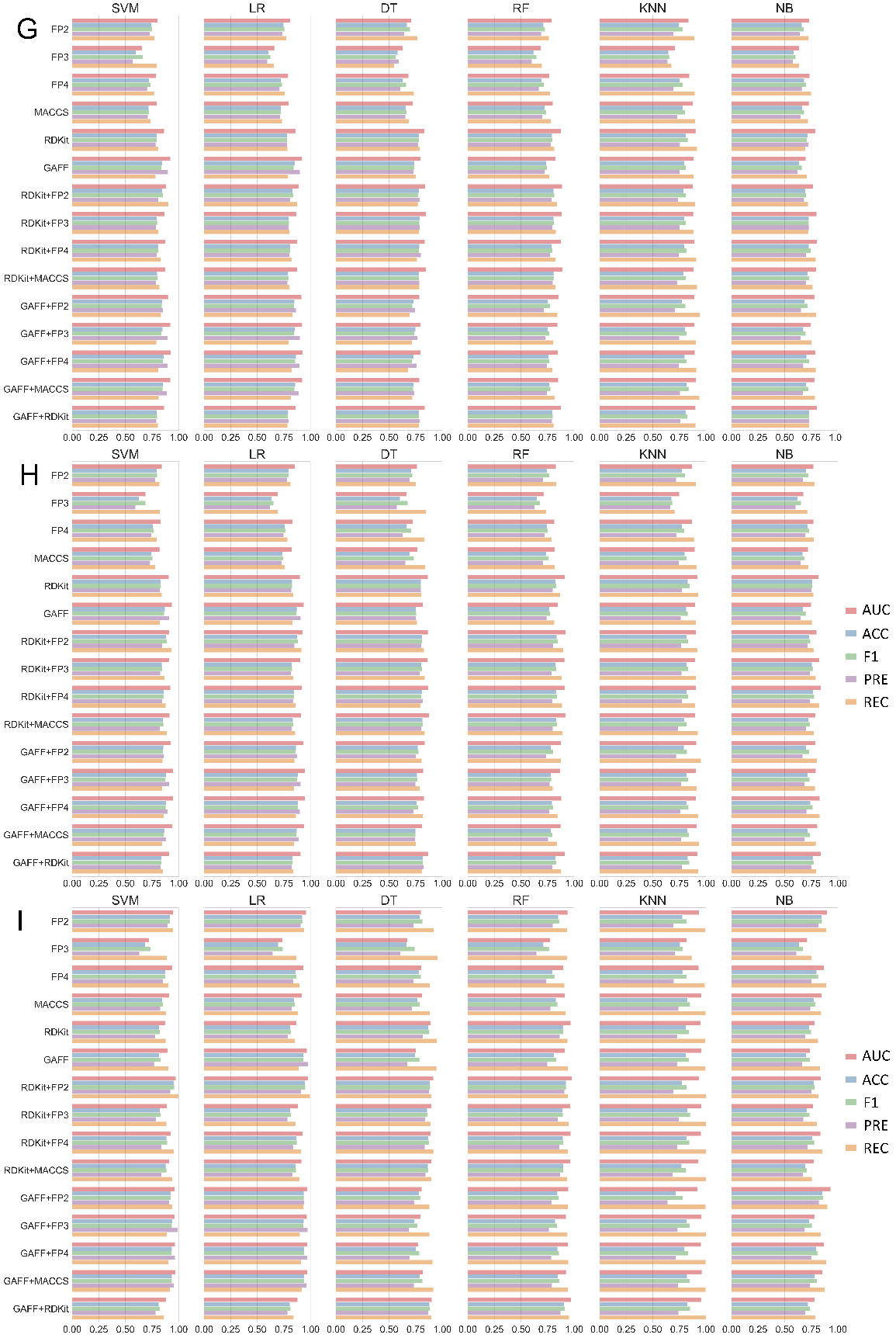
**

**Figure S1C** Model performance of validation sets for screening assays. G. AlphaLISA, H. TruHit, I. TMPRSS2.

**
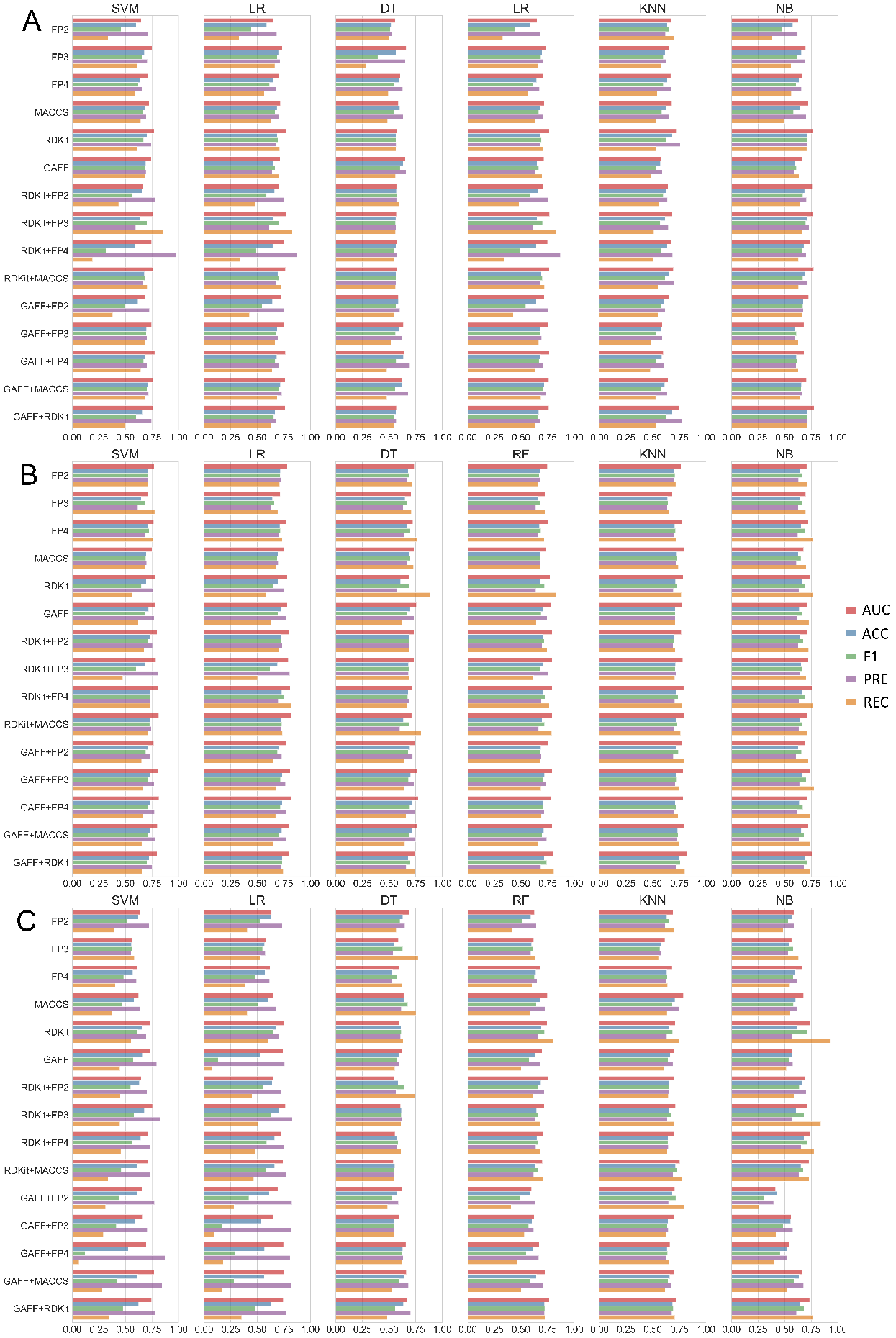
**

**Figure S2A** Model performance of test sets for screening assays. A. 3CL, B. HEK293, C. Fibroblast.

**
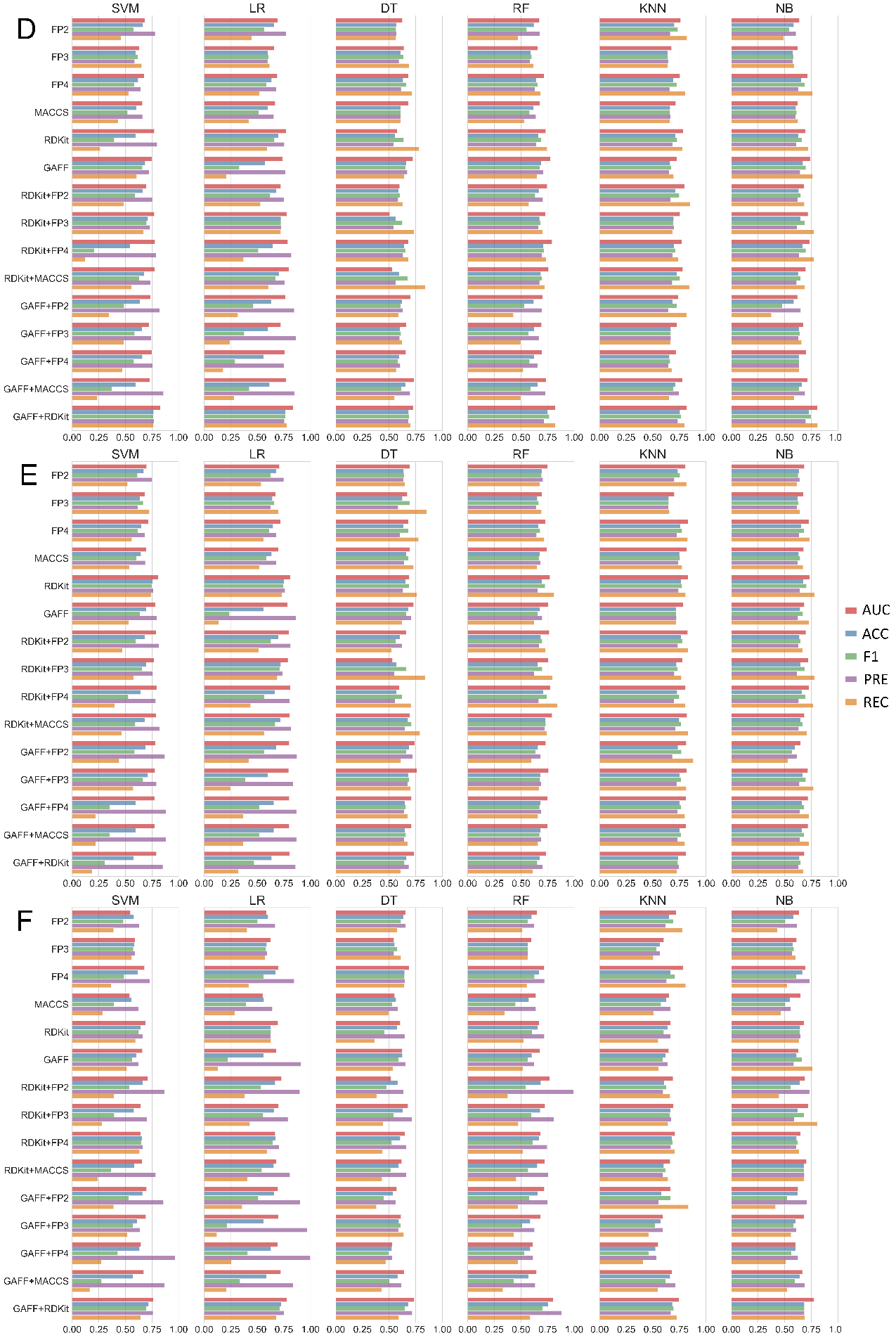
**

**Figure S2B** Model performance of test sets for screening assays. D. CPE, E. cytotox, F. ACE2.

**
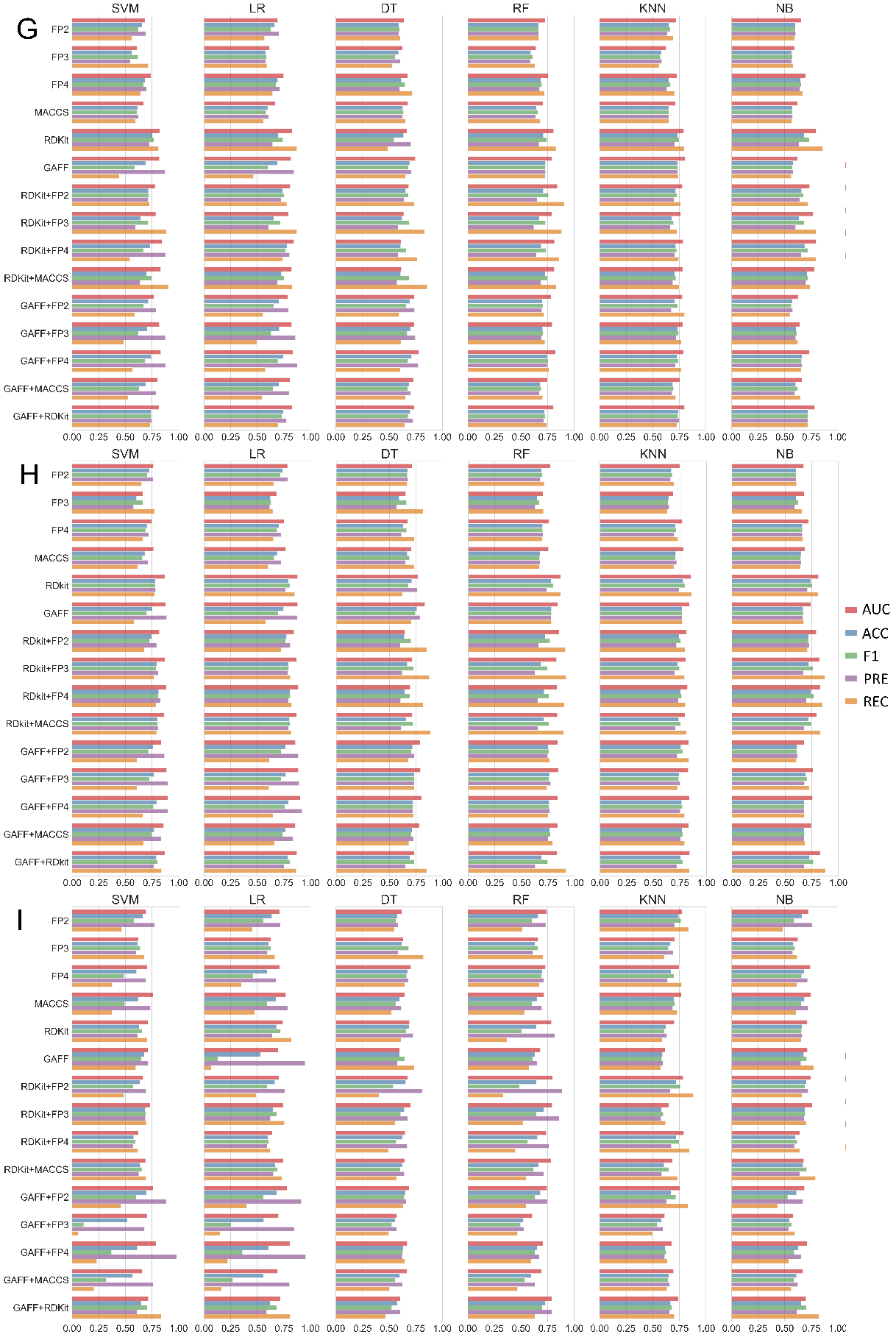
**

**Figure S2C** Model performance of test sets for screening assays: G, AlphaLISA; H, TruHit; I, TMPRSS2.

**
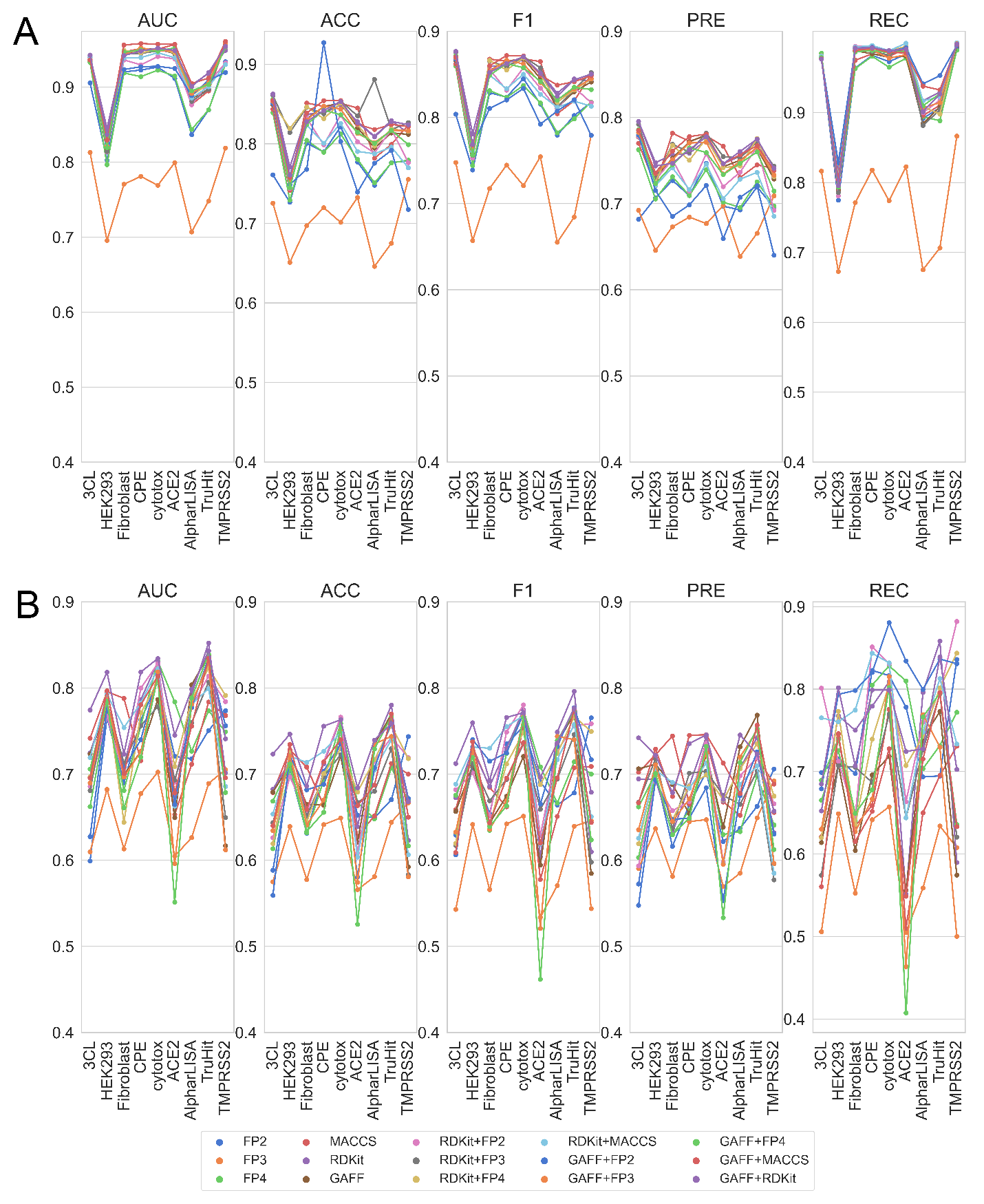
**

**Figure S3** metrics AUC, ACC, F1, PRE and REC for KNN models of validation set (A) and test set (B) using different molecular descriptors.


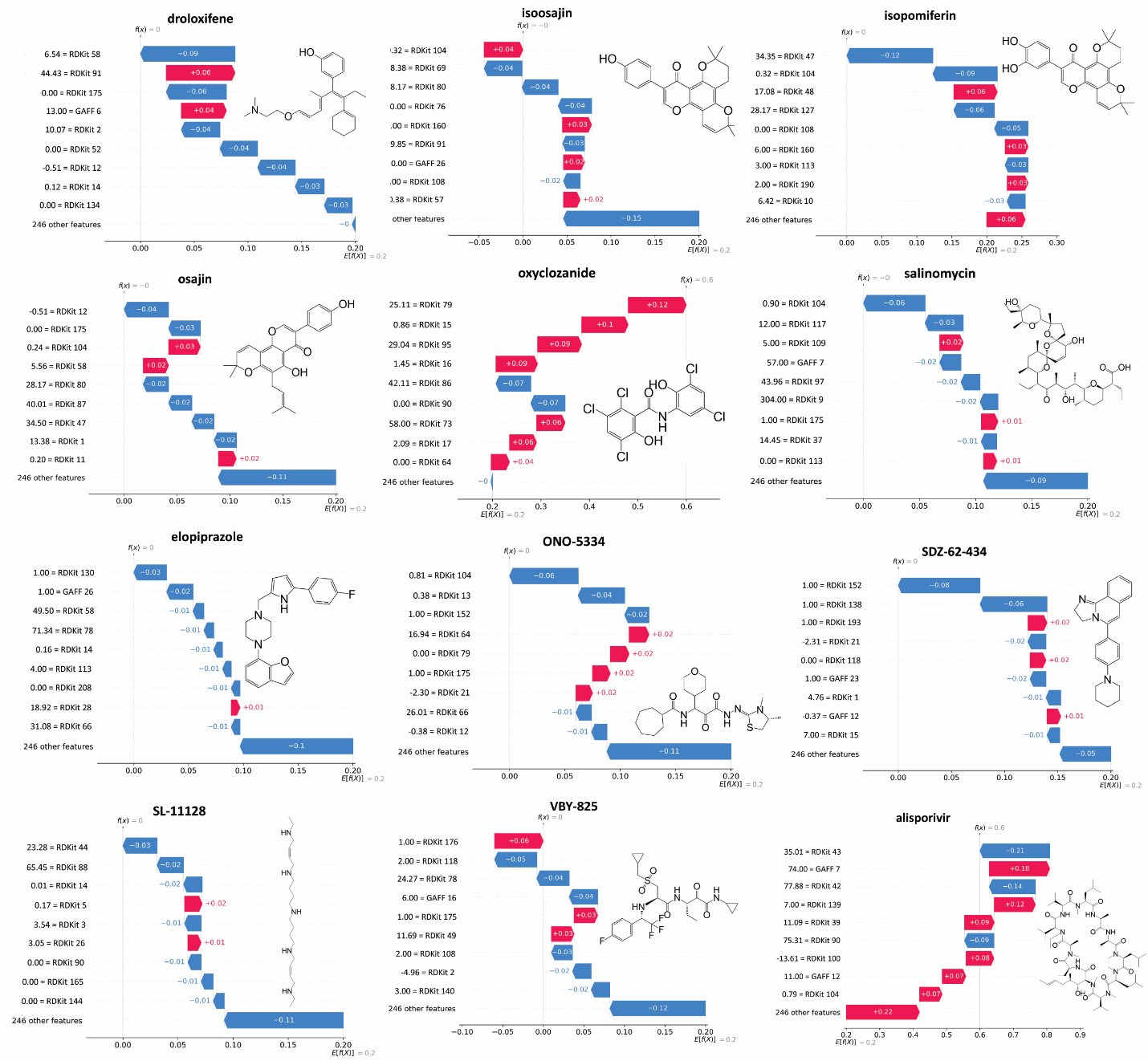


**Figure S4A** Importance of top 9 molecular features and the SHAP values for the first half of compounds in external CPE dataset.


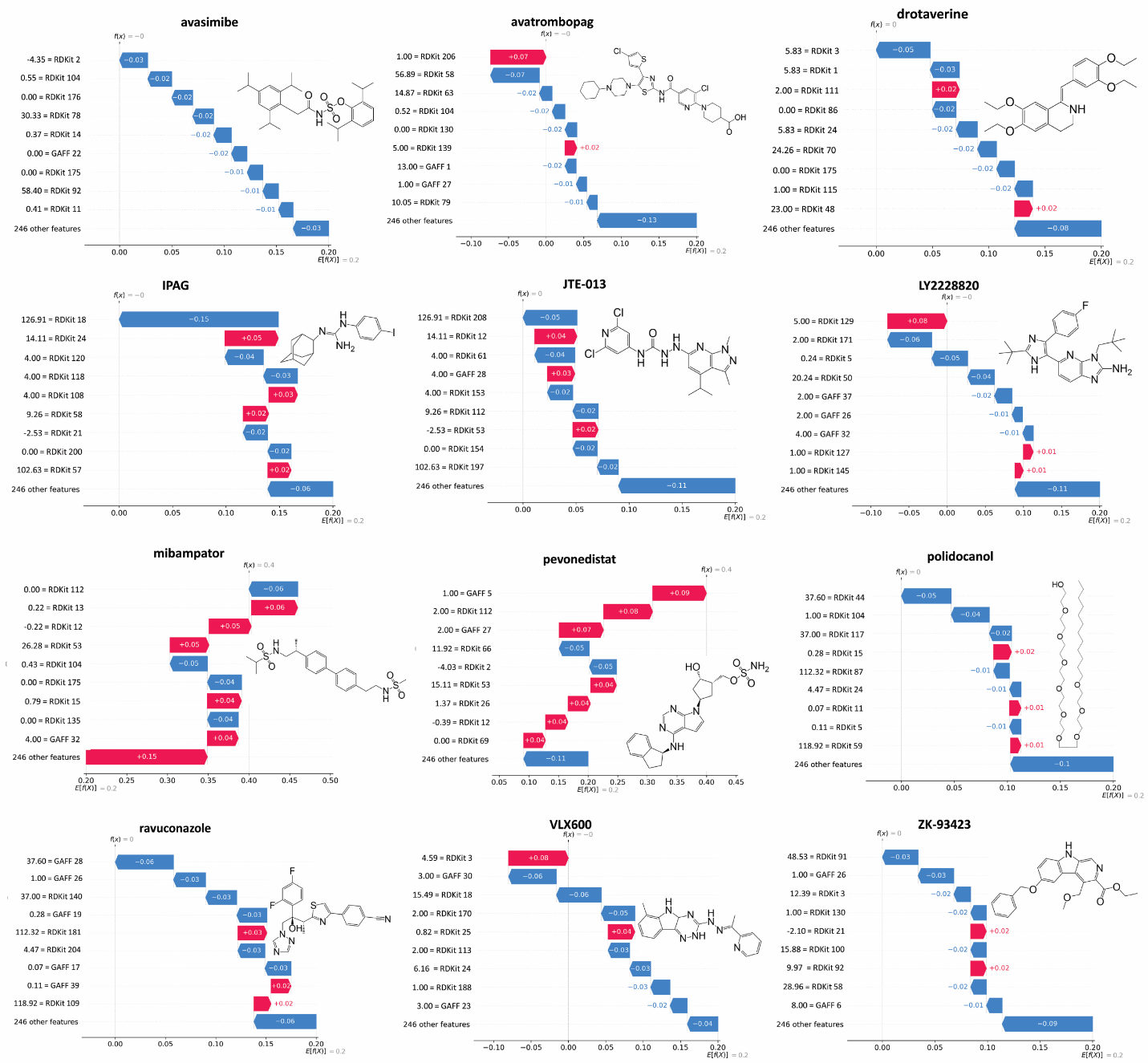


**Figure S4B** Importance of top 9 molecular features and the SHAP values for the second half of compounds in external CPE dataset.

**
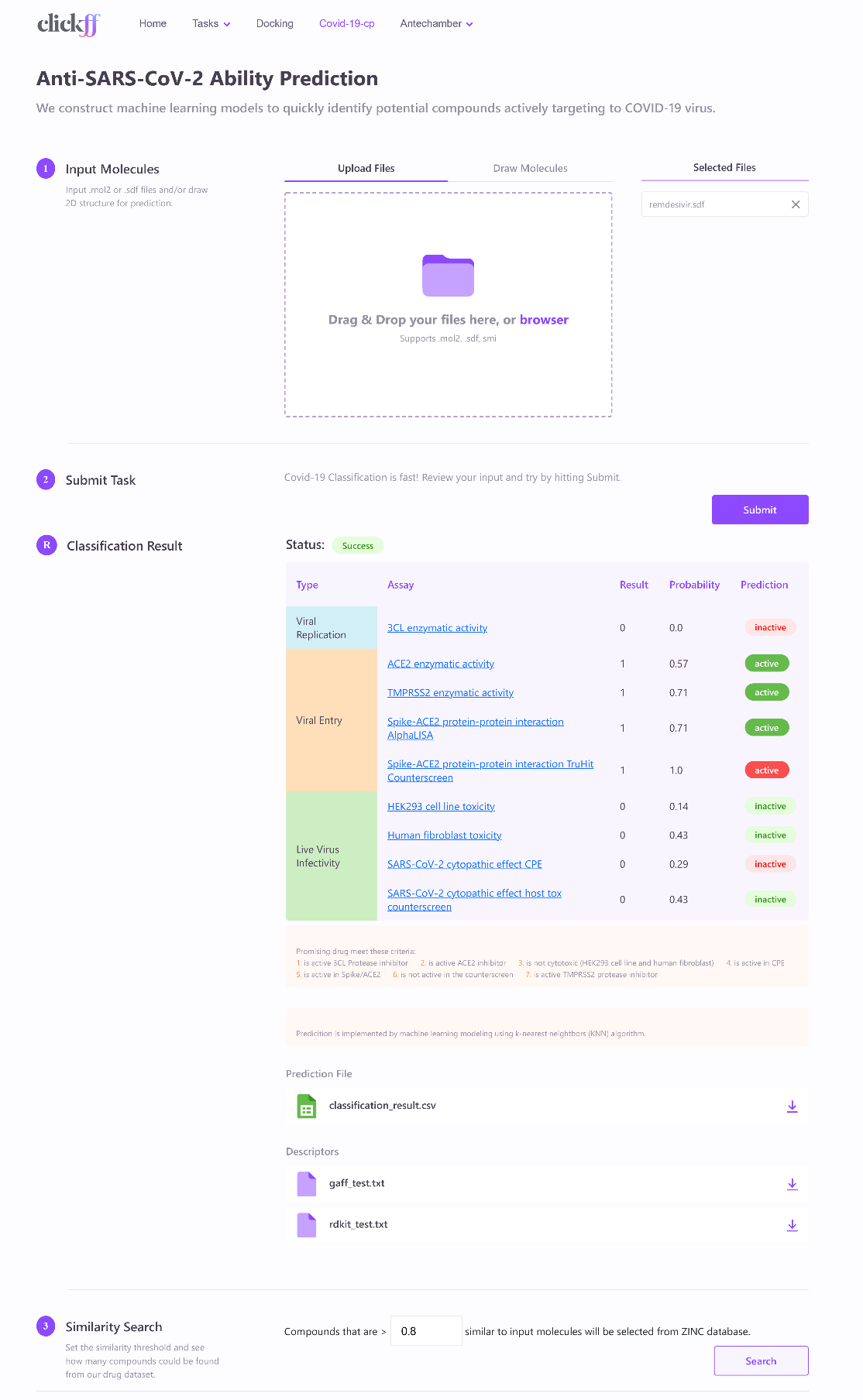
**

**Figure S5** User interface of the web portal.
